# Aerobic exercise interventions for older adults with cognitive frailty: a systematic review

**DOI:** 10.3389/fnagi.2026.1747309

**Published:** 2026-06-25

**Authors:** Xu Zhou, Lin Chen, Zheng Huang, Zihao Zhang, Meng Li, Zhongfang Yang

**Affiliations:** 1 Department of Orthopaedics, Shanghai Municipal Hospital of Traditional Chinese Medicine, Shanghai University of Traditional Chinese Medicine, Shanghai, China; 2 Nursing Department, Shanghai Municipal Hospital of Traditional Chinese Medicine, Shanghai University of Traditional Chinese Medicine, Shanghai, China; 3 School of Nursing, Gansu University of Chinese Medicine, Lanzhou, Gansu, China; 4 School of Nursing, Suzhou Medical College of Soochow University, Suzhou, China

**Keywords:** aerobic exercise, Chinese traditional medicine, cognitive function, frailty, older adults

## Abstract

**Objective:**

The purpose of this study was to systematically review the effects of aerobic exercise on cognitive function in older adults using randomized controlled trials (RCTs).

**Design:**

This study was a systematic review.

**Data sources:**

Ten databases, namely PubMed, the Cochrane Library, Wanfang, China National Knowledge Infrastructure (CNKI), SinoMed, Medline, Embase, the Social Science Citation Index (SSCI), Bielefeld Academic Search Engine (BASE), and OpenGrey, were searched from their inception to 30 April 2026.

**The qualification standard of the selective study:**

The randomized controlled trials of the aerobic exercise interventions for the cognitive function of older adults were addressed.

**Results:**

A total of 16 studies were included in the review. The primary conclusion of the quoted studies was that they evaluated the effects of interventions on cognitive function. Many studies (68.8%) used a single-blinded design, while 6.3% used a double-blind design and 25% used an open-label design. Overall, 14 studies (87.5%) reported significant cognitive benefits. Interventions were classified as pure aerobic (*n* = 7), intrinsic/embedded (*n* = 6), simultaneous dual-task (*n* = 2), and sequential (*n* = 1) interventions. All pure aerobic and dual-task interventions improved cognition; the single sequential trial showed no cognitive gain. Intrinsic/embedded interventions consistently enhanced both cognitive and physical outcomes. Two studies (12.5%) found no overall cognitive improvement, but one reported improvements in male patients. Intensity monitoring was heterogeneous. Quality assessment indicated a low risk of bias in a majority of trials.

**Conclusion:**

These findings prove that aerobic exercise benefits cognition, verbal fluency, attention, sleep quality, executive function, flanker task reaction time, and postural balance. Simultaneous dual-task aerobic exercise may yield larger executive and memory gains than lower-cognitive-load activities.

**Systematic review registration:**

PROSPERO, CRD420251061109.

## Highlights

What is already known on this topic

- Population aging is a global trend that is accompanied by increased prevalence of multimorbidity and a growing need for dementia-related care.
- Cognitive frailty is a potentially reversible geriatric syndrome.
- Cognitive frailty is considered a priority target for preventive interventions in healthcare systems.

What this study adds

- Aerobic exercise consistently improved global cognition, memory, executive functions, and physical outcomes in older adults with cognitive frailty.
- Different modalities of aerobic exercise were not equally effective.
- The findings were supported by biological signals of neuroprotection observed in some included trials, suggesting potential underlying mechanisms.

## 1 Introduction

Population aging is accelerating worldwide and is accompanied by increased prevalence of multimorbidity, functional dependence, and dementia-related care needs among older adults (Jenkins et al., 2023). Cognitive frailty has emerged as a clinically salient yet potentially reversible geriatric syndrome. Older adults with cognitive frailty experience higher rates of disability, falls, hospitalization, and mortality and face faster trajectories toward dementia and loss of independence (You et al., 2021; Cheng et al., 2025; Chen et al., 2025). The global prevalence of cognitive frailty among community-dwelling older adults is approximately 12.2% (Zhang et al., 2024). In China, the overall prevalence has been reported to be 15%, with higher rates observed among women than among men and in institutional or hospital settings compared with community settings (Liu et al., 2023; Huang et al., 2023). In addition, the prevalence has increased in recent years (Wang et al., 2025). These features render cognitive frailty a priority target for prevention and early intervention within primary care and community health systems.

Interventions for cognitive frailty include pharmacological and non-pharmacological interventions. However, the use of medications faces major challenges. On the one hand, polypharmacy can lead to pharmacodynamic and pharmacokinetic interactions that reduce therapeutic efficacy (Yu et al., 2024; Dixe et al., 2023; Kim and Kim, 2024). On the other hand, proper medication management requires cognitive abilities that these patients often lack (Kim and Kim, 2024; Ibrahim et al., 2024). These limitations highlight the urgent need for effective, low-risk, and cognitively accessible non-pharmacological interventions. Cognitive frailty is the concurrence of physical frailty and mild cognitive impairment (Kocyigit et al., 2024). Cognitive frailty involves bidirectional brain–body interactions, including sarcopenia, reduced cardiorespiratory fitness, inflammation, metabolic dysregulation, cerebrovascular compromise, and mood and sleep disturbances (Diniz et al., 2022). These characteristics highlight multiple modifiable pathways of lifestyle interventions, particularly structured physical activity.

Aerobic exercise refers to continuous physical activity involving large muscle groups (Thompson et al., 2013). Common examples include walking, jogging, cycling, stepping, and dancing. Aerobic exercise is the most accessible and scalable modality, requiring minimal equipment and being feasible in community and home-based settings. Scholars recommend aerobic training for both fit and frail older adults (Izquierdo et al., 2021). It should be part of daily exercise to reduce the risk of chronic diseases (Izquierdo et al., 2021). Aerobic exercise directly improves cardiorespiratory fitness and vascular health. These determinants are closely related to cognitive and physical performance. Moreover, aerobic exercise enhances prefrontal-hippocampal functional connectivity, improves cognitive control, and reduces global brain atrophy (Xia et al., 2025; Huang et al., 2024). Aerobic exercise has become a primary non-pharmacological option for cognitive frailty.

Despite these established benefits, several controversies regarding aerobic exercise for cognitive function persist. First, the comparative efficacy of different exercise modalities varies considerably (Zang et al., 2026). Second, the effect of exercise intensity on cognitive outcomes remains unclear, as previous studies have reported inconsistent findings (Izquierdo et al., 2021; Ichige et al., 2026; Park, 2022). Third, the extent to which the cognitive benefits of aerobic exercise are mediated by improvements in cardiorespiratory fitness remains debated (Tari et al., 2025; Dhahbi et al., 2025). Accordingly, this review uniquely focuses on four distinct modalities: pure aerobic, sequential, simultaneous dual-task, and intrinsically embedded interventions. It aims to clarify the roles of motor complexity, cognitive load, and temporal structure in driving cognitive improvement. This will help inform evidence-based exercise prescriptions.

Over the past 10 years, aerobic exercise trials in older adults with cognitive impairment have increased substantially. Many of these trials have been conducted in Asian countries. In resource-limited environments, understanding effective measures is essential. Embedding them into age-friendly community programs has direct implications for equity and scalability. Several systematic reviews have proven the cognitive benefits of aerobic exercise in older adults with mild cognitive impairment (Facal et al., 2019; Jia et al., 2025; Peng et al., 2024; Gates et al., 2013; Yuan et al., 2025; Li et al., 2025). However, they remain insufficient.

To the best of our knowledge, this is not the first systematic review on this topic. However, previous reviews have not systematically compared different aerobic exercise modalities based on how cognitive tasks are integrated with physical activity. First, previous reviews have discussed diverse intervention approaches. These include pure aerobic exercise, sequential exercise-cognitive training, simultaneous dual-task training, and intrinsically cognitively enriched modalities. However, they have not systematically compared the cognitive effects of these different modalities (Yuan et al., 2025). Second, the distinction between motor complexity and explicit cognitive load has not been addressed in these reviews. Evidence has indicated that these factors may engage distinct neural pathways (Li et al., 2025). Third, it remains unclear whether the temporal structure of cognitive and physical stimuli moderates neuroplastic effects. Notably, the only sequential trial in this review reported no cognitive benefits despite improved physical function.

To address these gaps, we conducted a systematic review of aerobic exercise interventions for older adults with cognitive frailty. Our objectives are as follows:

- To synthesize evidence from studies targeting cognitive frailty;
- To compare the efficacy of different aerobic exercise modalities;
- To distinguish between motor complexity and cognitive load as potential drivers of cognitive improvement; and.
- To examine the temporal relationship between cognitive and physical stimuli as a moderator of neuroplastic effects.

## 2 Methods

This review was conducted and registered with the Preferred Reporting Items for Systematic Reviews guidelines. It was also registered with the International Prospective Register of Systematic Reviews (PROSPERO: CRD420251061109). Our team includes authors with abundant expertise in gerontology, focusing on the research, development, implementation, and evaluation of fall prevention interventions and guidance, thereby ensuring the relevance and contextual insight of this review.

### 2.1 Search strategy

Ten electronic databases were systematically searched in Chinese and English from their inception to 30 April 2026. Peer-reviewed literature was retrieved from PubMed, the Cochrane Library, Wanfang, CNKI, SinoMed, Medline, Embase, the Social Science Citation Index (SSCI), Bielefeld Academic Search Engine (BASE), and OpenGrey databases, using a comprehensive set of terms for aerobic exercise interventions for older adults with cognitive frailty. The study design and specific subject headings of each database (Table 1) were adopted. The “AND” and “OR” operators were used to combine terms and conditions. There are no restrictions on the publication year. Experts in the medical industry were identified by studies quoted in the initial search and the author’s knowledge and were also contacted to recommend other relevant articles. The reference lists of relevant systematic reviews were also searched to obtain additional articles.

**Table 1:** Design characteristics.

Author, year, country	Methods for generating random sequences	Random allocation methods	Allocation concealment (Yes/No)	Blinding	Blinded parties	Follow-up		Sample size calculation and justificatior	Control group activity type	Assessment tool	Data processing methods
						Number of follow-ups	Duration of follow up				
Choi and Lee (2018), South Korea	Computer-generated randomization	Simple randomization	No	Single-blind	Outcome assessors	1	8 weeks	Effect size	Perform a home exercise program	Questionnaire, physiological measurement	ITT
Krootnark et al. (2024), Thailand	Computer-generated randomization	Stratified randomization	Sealed envelope method	Single-blind	Outcome assessors	1	3 months	Effect size	Daily activities	Questionnaire	ITT
Suzuki et al. (2013), Japan	Computer-generated randomization	Stratified randomization	No	Single-blind	Outcome assessors	1	6 months	Statistical power	Two education classes	Questionnaire	PP
ten Brinke et al. (2015), Netherlands	Computer-generated randomization	Simple randomization	Yes	Single-blind	Outcome assessors	1	26 weeks	Effect size	Balance and tone training	Physiological measurement	PP
Zotcheva et al. (2022), Norway	Computer-generated randomization	Simple randomization	No	Single-blind	Outcome assessors	3	5 years	/	Unsupervised control	Questionnaire, physiological measurement	PP
Wang et al. (2019), China	Random number table method	Simple randomization	No	Open-label	/	1	3 months	/	Education	Questionnaire	PP
Xu et al. (2023), China	Random number table method	Simple randomization	No	Open-label	/	1	3 months	/	Routine care and health education	Questionnaire	PP
Yao et al. (2024), China	/	/	No	Open-label	/	1	6 months	/	Routine care and health education	Questionnaire, physiological measurement	ITT
Choi and Lee (2019), Republic of Korea	Computer-generated randomization	Simple randomization	No	Single-blind	Outcome assessors	1	8 weeks	Effect size	Perform home exercises.	Questionnaire, physiological measurement	ITT
De Sá et al. (2024), Brazil	Random number table method	Block and stratified randomization	Sealed envelope method	Double-blind	Outcome assessors and researcher	1	6 months	/	Participated in a multimodal physical exercise protocol	Questionnaire, physiological measurement	PP
Hsu et al. (2018), Canada	Computer-generated randomization	Simple randomization	Yes	Single-blinded	Outcome assessors	1	6 months	/	Usual care education	Questionnaire, physiological measurement	PP
Li et al. (2023), USA	Computer-generated randomization	Block randomization	No	Single-blinded	Outcome assessors	3	48 weeks	Effect size	Stretching exercise	Questionnaire, physiological measurement	PP
Makino et al. (2021), Japan	Minimization algorithm	Stratified randomization	Yes	Single-blinded	Outcome assessors	2	52 weeks	Effect size	Educational classes	Questionnaire, physiological measurement	ITT
Silva et al. (2025), Portugal	/	Simple randomization	/	Open-label	/	1	12 weeks	Effect size	Strength training	Questionnaire, physiological measurement	PP
Song and Yu (2019), China	Computer-generated randomization	Block randomization	Sealed envelope method	Single-blinded	Outcome assessors	1	16 weeks	Effect size	Health education programme	Questionnaire	ITT
Chen et al. (2023), China	Computer-generated randomization	Block randomization	Yes	Single-blinded	Outcome assessors	2	36 weeks	/	Maintained their previous lifestyle.	Questionnaire, physiological measurement	ITT, PP

CG, control group; DASC, dementia assessment sheet in community-based integrated care system; IG, intervention group; MMSE, mini-mental state examination; RCT, randomized controlled trial; TMT, Trail Making Test.

### 2.2 Operationalization of selection criteria

Cognitive frailty is an emerging concept formally defined in 2013 by the International Academy on Nutrition and Aging (IANA) and the International Association of Gerontology and Geriatrics (IAGG) as the simultaneous presence of physical frailty and mild cognitive impairment (MCI) in the absence of dementia. Cognitive frailty is still a contested and evolving concept. The key challenges are how to determine its operational definition and select appropriate assessment tools. Moreover, the criteria used to define cognitive frailty vary across studies (Varrasi et al., 2024). Cognitive frailty is a distinct domain that is independent of physical frailty (Gong and Zhang, 2023). We adopted a pragmatic operational definition for this systematic review. We included articles that examined the effects of aerobic exercise in older adults aged more than 60 years with cognitive impairment.

### 2.3 Inclusion criteria

Studies published in either English or Chinese were included. Participants were required to be aged 65 years or older in developed countries and 60 years or older in developing countries. Eligible studies met the definition of cognitive frailty, including reduced or impaired mental or intellectual function, cognitive impairment, disorder, decline, or dysfunction. The intervention involved aerobic exercise or an integrated program including aerobic exercise, such as walking, stair climbing, stretching, balance training, or the use of a recumbent arm–leg stepper. Only studies employing an experimental design, particularly randomized controlled trials, were included in this review.

### 2.4 Exclusion criteria

Review studies, letters, conference abstracts, and literature without full text were excluded from this review.

### 2.5 Study selection and data extraction

Zotero tools were applied to screen and remove the replicative studies. The literature screening was performed by four independent reviewers (XZ, ZY, LC, and ZZ). Two sets of 20 references were initially selected for double screening to guarantee the reliability and relevance. All reviewers discussed the problems they identified, fine-tuned, and finalized the inclusion and exclusion criteria. Following the group discussion, all reviewers re-screened the initial abstracts. The remaining abstracts were screened using the same procedure. An inter-rater reliability of more than 85% showed a high level of agreement among the reviewers. Two reviewers (XZ and ZY) screened randomized controlled trials (RCTs) involving aerobic exercise. The full-text reports of potentially eligible studies were subsequently evaluated by other reviewers. Reviewers then extracted the study and summarized the intervention features on a shared spreadsheet using a pre-piloted data extraction form. The extracted information included (i) author, year, and country; (ii) type of study; (iii) type of participants; (iv) number of participants; (v) age; (vi) gender; (vii) setting; (viii) intervention and follow-up period; and (ix) the outcome of the study.

### 2.6 Classification of exercise interventions

We classified aerobic exercise interventions into four categories based on how cognitive tasks are integrated with physical activity: (i) Pure aerobic exercise: These interventions involved only continuous, rhythmic aerobic activity (e.g., walking, stepping, and jogging), without any cognitive or complex motor skill training (Talar et al., 2022). (ii) Sequential intervention: Cognitive training (e.g., computer-based memory or executive tasks) was delivered separately after the aerobic session, with no temporal integration between the two components (Cherbuin et al., 2026). (iii) Simultaneous dual-task intervention: Participants performed an explicit cognitive task concurrently with aerobic exercise (e.g., walking while counting backward), thereby engaging attentional and executive resources (Falbo et al., 2016; Trombini-Souza et al., 2023). (iv) Intrinsically embedded intervention: These interventions involve learning and executing complex motor skills (e.g., Taichi, Baduanjin, and kayak paddling), which inherently engage cognitive processes such as attention, memory, and motor planning. No secondary cognitive task is added (Liu et al., 2026).

### 2.7 Data extraction: exercise intervention characteristics

To characterize the exercise interventions across studies, two reviewers independently extracted data based on the following predefined dimensions: (i) intervention type, defined according to how cognitive tasks were integrated with physical exercise. Based on a preliminary review of the literature, this classification was developed as a priority and included four categories: (a) pure aerobic exercise—interventions involving only aerobic activity without any cognitive training component; (b) sequential interventions—interventions where cognitive training was delivered separately after the exercise session; (c) simultaneous dual-task interventions—interventions requiring participants to perform cognitive tasks concurrently with exercise; and (d) intrinsic/embedded interventions—interventions involving complex motor skills (e.g., Taichi, Baduanjin, and kayak paddling) that inherently engage cognitive processes such as attention, memory, and coordination without the addition of an explicit secondary cognitive task. (ii) Exercise parameters, including intervention duration (weeks), frequency (sessions per week), session length (minutes), and exercise setting (supervised group, home-based, or hybrid). (iii) Exercise intensity, including whether the study specified a target intensity and monitoring method. The intensity report was categorized as: (a) objectively monitored (e.g., heart rate monitors and ergospirometry); (b) subjectively monitored (e.g., the Borg rating of perceived exertion scale); or (c) not reported. Discrepancies between reviewers were resolved through discussion or consultation with a third reviewer. A summary of these extracted characteristics for each included study is presented in Supplementary Table S4.

### 2.8 Quality assessment

Quality assessment was conducted according to the Joanna Briggs Institute (JBI) critical appraisal criteria. Each included study was evaluated for its design, conduct, analysis, interpretation, and report to minimize systematic error. The rigor and quality of the studies were assessed using the JBI Critical Appraisal Checklist for Randomized Controlled Trials (JBI RCT Checklist). The criteria of assessment include (i) random sequence generation, (ii) allocation concealment, (iii) blinding of participants and personnel, (iv) blinding of outcome assessment, (v) incomplete outcome data, (vi) selective reporting, and (vii) other bias. An individual risk-of-bias risk assessment was not conducted due to the integrity and all-sidedness of the studies included in the Cochrane Collaboration. The risk of publication bias may arise when systematic reviews only included published studies, potentially resulting in the overestimation of the intervention’s effectiveness. Our systematic review attempted to mitigate such bias by incorporating the gray literature, thereby reducing the bias effects of the selective publication and providing a better understanding of the evidence. As a result, an individual assessment of publication bias was not conducted, either.

### 2.9 Statistical analysis and systematic synthesis

The intervention categories were designed and developed in terms of the ones evaluated in the quoted studies, and the conclusions were summarized and presented to align with each category. The included studies were characterized in terms of the types of aerobic exercise, the outcomes of cognitive function-related, the tracing period, and the effectiveness of cognitive function. Studies that assessed the behavioral outcome were also reviewed. If any inconsistent findings across samples or follow-ups were found, the interventions were classified as having mixed results.

The included studies showed the heterogeneity in study designs, interventions, and outcomes; therefore, Synthesis Without Meta-analysis (SWiM) was applied to exhibit the data synthesis by descriptive vote counting and grouping the included studies. Based on the effect direction, vote counting compared the numbers of the studies with and without improved outcomes. The magnitude of effect was not recorded and reported due to the diversity and complexity of the designs, interventions, and outcomes of the included studies. Moreover, a standard binary metric (benefit or mixed results) was designed to calculate the proportion, which is 95% confidence interval (CI) (binomial exact calculation) and *p*-value (binomial probability test), and indicate the effectiveness of each intervention category across the outcomes measured.

### 2.10 Commitment to equity, diversity, and inclusion

The team of investigators, although exclusively based in China, is gender-balanced and includes student and senior researchers from various disciplines. The study populations encompassed individuals from diverse socioeconomic and cultural backgrounds, including older adults with cognitive frailty from low- and middle-income countries (LMICs).

### 2.11 Patient and public engagement

In this systematic review, patients and the public were not engaged.

## 3 Results

### 3.1 Overview

**Figure 1:**
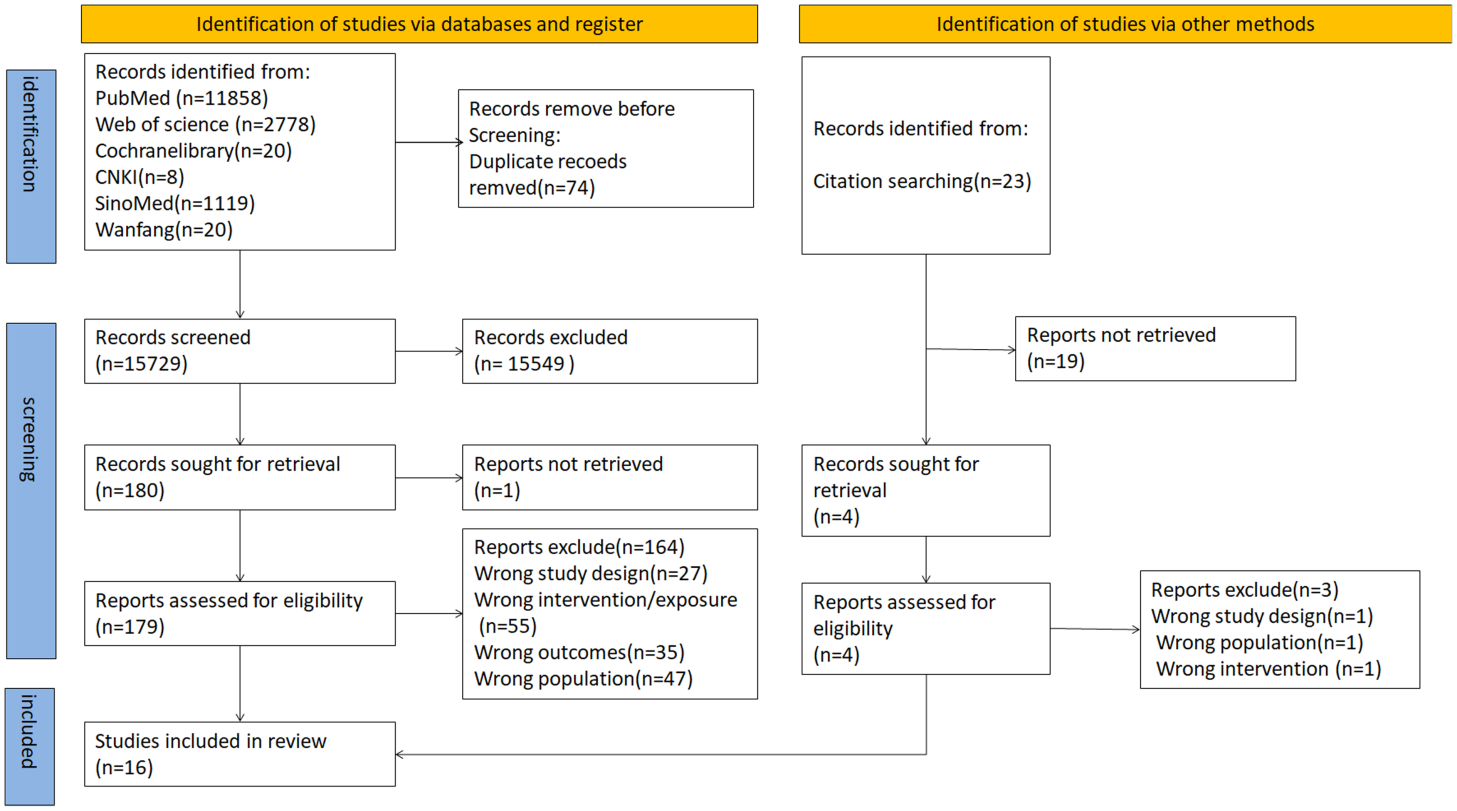
Preferred reporting items for systematic reviews flowchart. RCTs, randomized controlled trials.

A total of 15,803 studies were reviewed after the database search, and 74 reference lists were located based on the expert recommendations, gray literature, and other relevant reference checking. Overall, 15,729 studies were screened in accordance with the inclusion criteria. After duplicates were removed, 15,713 full-text studies were retrieved for final qualification review. Eventually, 16 studies were included (Figure 1), and all included studies are referenced in the Supplementary material (Tables 1, 2, Supplementary Tables S1–S5).

**Table 2:** Characteristics of the included studies.

Author, year, country	Type of study	Type of participants	Number of participants	Age (year)	Gender (female, %)	Setting	Intervention	Follow-up period	Diagnostic criteria	Cognitive domains assessed
Choi and Lee (2018), South Korea	RCT	Older adults with MCI	IG:30	IG:74.9 ± 5.1 yrs.	IG: 80	Senior welfare center	IG: Ground Kayak Paddling Exercise twice a week for 6 weeks on the ground, and each session consisted of 10 min of warm-up activities, 40 min of GKP exercise, and 10 min of cool-down	8 weeks	MoCA	<6 points
			CG:30	CG:74.23 ± 4.38 yrs	CG: 83.3		CG: Home exercise program twice a week for 6 weeks and received a weekly confirmation call from the instructor			
Krootnark et al. (2024), Thailand	RCT	Older adults with MCI	The aerobic exercise group:30	The aerobic exercise group: 68.60 ± 4.86 yrs.	The aerobic exercise group:80	Community	The aerobic exercise group: Low impact exercise: indoor walk, march in place, step in difference directions	3 months	MoCA (Thai version)	17–24 points
			The resistance exercise group:30	The resistance exercise group: 68.70 ± 4.72 yrs.	The resistance exercise group:76.67		The resistance exercise group: Shoulder flexion, abduction, elbow flexion, extension, hip extension, abduction, Knee extension, plantar flexion, wall push up, step ups			
			CG:30	CG:69.70 ± 5.55 yrs	CG:80		CG: continue their usual daily life activities with the exception of engaging in any type of exercise or cognitive training until the end of the study			
Suzuki et al. (2013), Japan	RCT	Older adults with MCI	IG: 50 IG: 50	IG: 74.8 ± 7.40 yrs.	IG: 50	Community	IG: biweekly 90-min sessions involving aerobic exercise, muscle strength training, postural balance retraining, and dual-task training, a focus on promoting exercise and behavior change	6 months	MMSE; ADAS-cog; WMS-R Logical Memory	Petersen critreria; Memory score < 1.5 SD below age mean
			CG: 50	CG: 75.80 ± 6.10 yrs	CG: 48		CG: two education classes about health promotion involving healthy diet, oral care, prevention of urinary incontinence, and health checks			
ten Brinke et al. (2015), Netherlands	RCT	Older woman with MCI	Resistance training (RT):8	Resistance training (RT):73.75 ± 3.72 yrs.	AT:100	Community	RT: Keiser Pressurised Air system(biceps curls, triceps extension, seated row, latissimus dorsi pull downs, leg press, hamstring curls and calf raises) + other key strength exercises included mini-squats, mini-lunges and lunge walks. AT: outdoor walking programme	26 weeks	MoCA; MMSE	MoCA < 26 MMSE ≥ 24
			Aerobic training (AT): 10	Aerobic training (AT): 76.07 ± 3.43 yrs.	RT:100					
			Balance and tone (BAT):0.11	Balance and tone (BAT):. 75.46 ± 3.93 yrs	BAT:100		BAT:stretching exercises, range of motion exercises, balance exercises, functional and relaxation techniques. 60 min in duration daily			
Zotcheva et al. (2022), Norway	RCT	Older adult s	MICT:235	MICT: 72.3 ± 2.05 yrs.	MICT:48.5	Community	MICT: continuous training consisted of 50 min of continuous aerobic exercise at moderate intensity (70% of peak heart rate), corresponding to ~ 13 on the Borg scale for ratings of perceived exertion almost daily, or to two weekly sessions of aerobic exercise over 5 years.	5 years	MoCA	1.5 SD below age/education-specific norm
			HIIT:216	HIIT: 72.4 ± 2.08 yrs.	HIIT:44.9		HIIT: comprised ~ 40 min of interval training, consisting of 4-min working periods at 85–95% of peak heart rate (~ 16 on the Borg scale) (Facal et al., 2019) with 3-min active breaks (60–70% of peak heart rate) in between twice weekly			
			CG:494	CG: 72.2 ± 2.02 yrs	CG:48.4		CG: Recommended to follow national recommendations for physical activity			
Wang et al. (2019), China	RCT	Older adults with MCI	IG: 41	IG: 68.40 ± 7.4 yrs.	IG:60.7	Community	IG: gymnastic exercises + community usual health education	3 months	MoCA	15–24 points
			CG: 42	CG: 69.65 ± 7.18 yrs.	CG:62.5		CG: community usual health education			
Xu et al. (2023), China	RCT	Older adults with aMCI	IG: 31	IG:67.5 ± 7.3 yrs.	IG: 71	Hospital	IG: Routine care and health education +12-week practice of Baduanjin	3 months	MoCA; AVLT-H; MMSE	MoCA <26; MMSE education-adjusted (illiterate 17–19, primary 20–22, ≥middle school 23–26); memory score <1.5 SD below normal
			CG: 32	CG: 68.6 ± 7.5 yrs	CG: 69		CG: Routine care and health education			
Yao et al. (2024), China	RCT	Older adults with aMCI	IG: 32	IG:68. 48 ± 6. 14 yrs.	IG: 53.1	Hospital	IG: Routine care and health education +Six-month Baduanjin Combination of exercise and cup-stacking training	6 month	MoCA	MoCA education-adjusted
			CG: 30	CG: 68. 15 ± 5. 64 yrs	CG: 46.7		CG: Routine care and health education			
Choi and Lee (2019), Republic of Korea	RCT	Older adults with MCI	IG:30	IG: 77.27 ± 4.37 yrs.	IG:83.3	Welfare center	IG: VKP Exercise each session consisted of a 10-min warm-up, 40-min VKP exercise, and 10-min cooldown. The paddling exercise in a virtual environment for 60 min twice a week for 6 weeks.	6 weeks	MoCA	<26 points
			CG:30	CG: 75.37 ± 3.97 yrs	CG:86.7		CG: home exercise			
De Sá et al. (2024), Brazil	RCT	Older adults with MCI	Physical Exercises (PE):9	(PE):70.0 [61–82] yrs.	(PE):77.8	Geriatric clinics	PE: a multimodal physical exercise protocol, predominantly aerobic and comprising the work of the components of functional capacity	6 months	MMSE; CDR	CDR 0.5–1.0
			Motor Tasks (MT):9	(MT):73.6 [63–84] yrs.	(MT):77.8		MT: the motor task complexity protocol was based on the two-dimensional model of Gentile’s taxonomy PE + MT: formed with the intention of proposing activities that addressed both interventions			
			PE + MT:9	PE + MT:70.3 [64–83] yrs	PE + MT:77.8		Six months of intervention twice a week resulted in improvements in cognitive function, total cholesterol			
Hsu et al. (2018), Canada	RCT	Older adults with mild vascular cognitive	IG:10	IG: 71.7 ± 8.8 yrs.	IG: 60	Clinics	IG: Aerobic training and compliance thrice-weekly 60 min classes of walking for the 6-month intervention period.	6 months	MoCA	Clinical diagnosis of mild vascular cognitive
			CG:11	CG: 72.3 ± 8.8 yrs	CG: 63.6		CG: usual care		MMSE	
Li et al. (2023), USA	RCT	Older adults with MCI	Enhanced Tai Ji Quan: 105	Enhanced Tai Ji Quan: 76.00 ± 5.10 yrs.	Enhanced tai ji quan: 71.4	Community	Enhanced Tai Ji Quan: the standard tai ji quan + practice in dynamic tai ji quan forms interwoven concomitantly with a set of cognitively demanding activities	16 weeks (mid-intervention), 24 weeks (postintervention–primary endpoint), and	MoCA	CDR global ≤0.5
			Standard Tai Ji Quan: 107	Standard Tai Ji Quan: 75.90 ± 5.10 yrs.	Standard tai ji quan: 61.7		Standard Tai Ji Quan: Participants received verbal and visual instruction cues for sequential practice of 8 Tai Ji Quan forms		CDR	MMSE ≥24
			Stretching: 106	Stretching: 76.00 ± 6.10 yrs	Stretching: 67.0		Stretching: participants received stretching exercises		MMSE	MoCA mean 25.2–25.3 at baseline
Makino et al. (2021), Japan	RCT	Older adults with subjective memory complaints	Aerobic exercise training protocols AT:88 Resistance exercise training protocols	AT: 72.25 ± 4.56 yrs.	AT:47.1	Community	AT: 10–15 min of step-in-place exercises+10-15 min of a walking program +intervals for rest and monitoring rate.	52 weeks	WMS-R Logical MemoryΠ; MMSE	Amnesia defined by ADNI criteria; MMSE≥ 20
			RT:89 Combine exercise training protocols	RT: 72.33 ± 4.77 yrs.	RT:48.0		RT: elastic resistance training + bodyweight exercises			
			CT:97	CT: 72.61 ± 4.52 yrs.	CT:41.3		CT: combined the AT and RT programs			
			CG:88	CG: 72.10 ± 4.61 yrs	CG:51.4		CG: attend educational classes 2 times during the 26-week			
Silva et al. (2025), Portugal	RCT	Older adults with cognitive decline	STCT: 53	STCT:73.8 ± 7.1 yrs.	STCT, 48.2	Community	STCT, strength plus cognitive training	12 weeks	MoCA	<26 points
			ST: 22	ST:73.1 ± 4.4	ST, 73.9		ST, strength training			
			AT: 41	AT:71.9 ± 6.3	AT, 92.7		AT, aerobic training			
			ATCT: 34	ATCT:71.9 ± 4.9	ATCT, 73.5		ATCT, aerobic plus cognitive training The interventions were implemented over 12 consecutive weeks, comprising 60-min sessions conducted three times per week, with a 48-h interval between each session.			
Song and Yu (2019), China	RCT	Older adults with MCI	IG:60	IG: 76.22 ± 5.76 yrs.	IG:80	Community	IG: aerobic stepping exercise programme with three 60-min group training sessions (20 participants per group) per week	16 weeks	MoCA-C (Chinese version)	19–26 points (education-adjusted)
			CG:60	CG: 75.33 ± 6.78 yrs	CG:70		CG: 16-week health education programme			
Chen et al., 2023, 2023, China	RCT	Older adults with MCI	Tai chi chuan group: 107	Tai chi chuan group: 67.56 ± 4.99 yrs.	IG:54.2	Community	Tai chi chuan group: T2D management+ 24-form tai chi chuan training.	36 weeks	MoCA	Clinical diagnosis of MCI (no dementia); baseline MoCA mean 21.34–21.52
			Fitness walking group: 110	Fitness walking group: 67.46 ± 4.73 yrs.	Fitness walking group 44.5%		Fitness walking group: T2D management + 24-week fitness walking program.			
			CG:111	CG: 67.62 ± 5.35 yrs	CG: 54.1		CG: T2D management Both exercise groups took the training for 60 min/session, 3 times/wk., for 24 weeks in a supervised setting			

### 3.2 Characteristics of the included studies

The characteristics of the study settings and populations of the included studies are summarized in Table 2. The included research was predominantly conducted in the USA (*n* = 1, 6.25%), the Netherlands (*n* = 1, 6.25%), China (*n* = 5), South Korea (*n* = 1), the Republic of Korea (*n* = 1), Portugal (*n* = 1, 6.25%), and Japan (*n* = 2). Accelerated aging processes accounted for the largest proportion of studies (*n* = 7, 43.8%). Studies conducted in countries with significantly aging populations constituted a further 25% (*n* = 4) of the total. Only one study (6.25%) was conducted before 2015.

The primary outcomes in the remaining three studies (18.75%) targeted specific metrics such as executive function, postural balance, or neural efficiency, with cognitive function enhancement incorporated as a secondary measure. The majority of studies (87.5%, *n* = 14) primarily addressed cognitive function in elderly populations. Health-related quality of life represented the second most common area of research, comprising 56.25% (*n* = 9) of the studies, such as memory (*n* = 3, 18.75%), depressive mood (*n* = 2 12.5%), muscle performance (*n* = 4, 25%), verbal fluency, attention, sleep quality, and executive function (*n* = 3, 18.75%). The most common target groups were older adults with MCI (*n* = 10, 62.5%), older adults with aMCI (*n* = 2, 12.5%), older adults with subjective memory complaints, older adults with mild vascular cognitive impairment, and older adults with cognitive decline. Design characteristics are shown in Table 1 and Supplementary Tables S1–S3.

A single-blinded design was the most common methodology, which was used in 68.8% of the studies. In contrast, double-blind and open-label designs were used in 6.3 and 25% of the studies, respectively. Approximately three-quarters of the studies (*n* = 7, 43.8%) were conducted using ITT data processing methods.

### 3.3 Description of intervention

The details regarding the intervention format and delivery are contained within Supplementary Table S1. The vast majority of interventions were delivered face-to-face (*n* = 15, 93.8%), with only one study using an online format. Intervention of the control group had an education or exercise component (*n* = 14, 87.5%); however, only approximately 12.5% of studies recommended usual care or unsupervised exercise. The majority of intervention providers were exercise program instructors (*n* = 9, 56.3%), while 12.5% of studies reported interventions delivered by nurses or doctors. A higher proportion of studies evaluated cognitive function at pre-intervention and post-intervention (*n* = 15, 87.5%), but approximately one-third evaluated cognitive function at follow-up (*n* = 5, 31.3%). Aerobic exercise included ground kayak paddling exercise, indoor walking, marching in place, stepping in different directions, arm movement, squatting while stepping, outdoor walking, gymnastics, Taichi, and Baduanjin exercise.

### 3.4 Outcomes

Supplementary Table S2 shows the information on the effect direction of the included studies. The majority of studies (87.5% of studies, *n* = 14) found a statistically significant benefit for cognitive function in older MCI patients. Conversely, a small subset of studies (*n* = 2, 12.5%) failed to demonstrate a significant cognitive improvement. In one study, the data on MoCA scores and MCI incidence revealed no statistically significant cognitive benefits from the exercise intervention. However, a stratified analysis has indicated that exercise was strongly associated with improved global cognition and a reduced risk of MCI in men. The most primary endpoint was cognitive function (*n* = 14, 87.5%). The secondary outcomes included walking costs, memory, short-term delayed memory, static balance, physical fitness, and blood metabolic indices.

### 3.5 Characteristics of interventions

A total of 16 randomized controlled trials were included, with interventions lasting from 6 weeks to 5 years. Exercise modalities varied considerably and were categorized into four types: pure aerobic exercise (*n* = 7, 43.75%), intrinsic/embedded interventions (*n* = 6, 37.5%), simultaneous/dual-task protocols (*n* = 2, 12.5%), and sequential interventions (*n* = 1, 6.25%).

Pure aerobic exercise interventions.

A total of seven studies examined pure aerobic exercise interventions. Aerobic modalities included walkin (ten Brinke et al., 2015; Hsu et al., 2018; Li et al., 2023), stepping exercise (Song and Yu, 2019), aerobic gymnastics (Wang et al., 2019), and moderate- to high-intensity continuous or interval training (Zotcheva et al., 2022). One study specifically evaluated low-intensity home-based aerobic exercise (Krootnark et al., 2024). All pure aerobic interventions demonstrated cognitive benefits, particularly for memory and executive function, with mechanisms including increased hippocampal volume, improved neural efficiency, and enhanced cardiorespiratory fitness.

### 3.6 Sequential interventions

Only one study used a sequential design combining physical exercise with separate cognitive training (Silva et al., 2025). In this 12-week trial, participants completed aerobic or strength training followed by 20 min of computerized cognitive training using the Fit4Alz software. Despite improvements in physical performance, no significant cognitive benefits were observed. The lack of cognitive effects may reflect the relatively short intervention duration or the limited sensitivity of the cognitive assessment tool.

### 3.7 Simultaneous/dual-task interventions

Two studies implemented simultaneous dual-task protocols that integrated cognitive and motor tasks concurrently (Li et al., 2023; Suzuki et al., 2013). Suzuki et al. implemented a 6-month multicomponent exercise program incorporating dual-task training such as walking while inventing poems and performing cognitive tasks during ladder stepping. Li et al. evaluated a 24-week cognitively enhanced Taichi intervention that required participants to perform concurrent cognitive tasks (e.g., recalling movement sequences, responding to deliberate miscues, and switching between forms) during exercise. Both studies demonstrated significant improvements in global cognition and memory, with sustained effects at follow-up and evidence of reduced brain atrophy or improved neural efficiency.

### 3.8 Intrinsic/embedded interventions

Six studies evaluated interventions that inherently engage cognitive processes through complex motor skill learning without explicit secondary cognitive tasks. These included mind–body exercises such as Baduanjin (Xu et al., 2023; Yao et al., 2024), Taichi (Chen et al., 2023), motor tasks based on Gentile’s taxonomy (De Sá et al., 2024), and novel activities, including ground kayak paddling (Choi and Lee, 2018) and virtual reality-based kayak paddling (Choi and Lee, 2019). Intervention durations ranged from 6 to 24 weeks. While intensity was not often quantitatively specified, these interventions consistently improved cognitive function, physical performance, and, in some cases, metabolic biomarkers (e.g., reduced Tau protein and improved lipid profile). Notably, in some studies, these effects occurred without concurrent increases in Brain-Derived Neurotrophic Factor (BDNF) levels, suggesting alternative mechanisms such as enhanced neural efficiency or reduced neuroinflammation.

### 3.9 Exercise intensity

The majority of studies targeted “moderate-intensity” aerobic exercise. However, the definitions and monitoring tools varied significantly across studies. Some studies used objective measures such as heart rate monitors (e.g., Brinke et al., 2013; Hsu et al., 2018) or ergospirometry (Zotcheva et al., 2022), while others relied on subjective ratings of perceived exertion (RPE) (e.g., Silva et al., 2025; Makino et al., 2021). Notably, several studies, particularly those involving mind–body exercises such as Baduanjin (Xu et al., 2023; Yao et al., 2024) and Taichi (Chen et al., 2023), did not specify intensity targets or monitoring protocols. This heterogeneity in intensity prescription and monitoring represents a key methodological consideration when interpreting the comparative effectiveness of different exercise modalities.

### 3.10 Quality assessment

**Figure 2:**
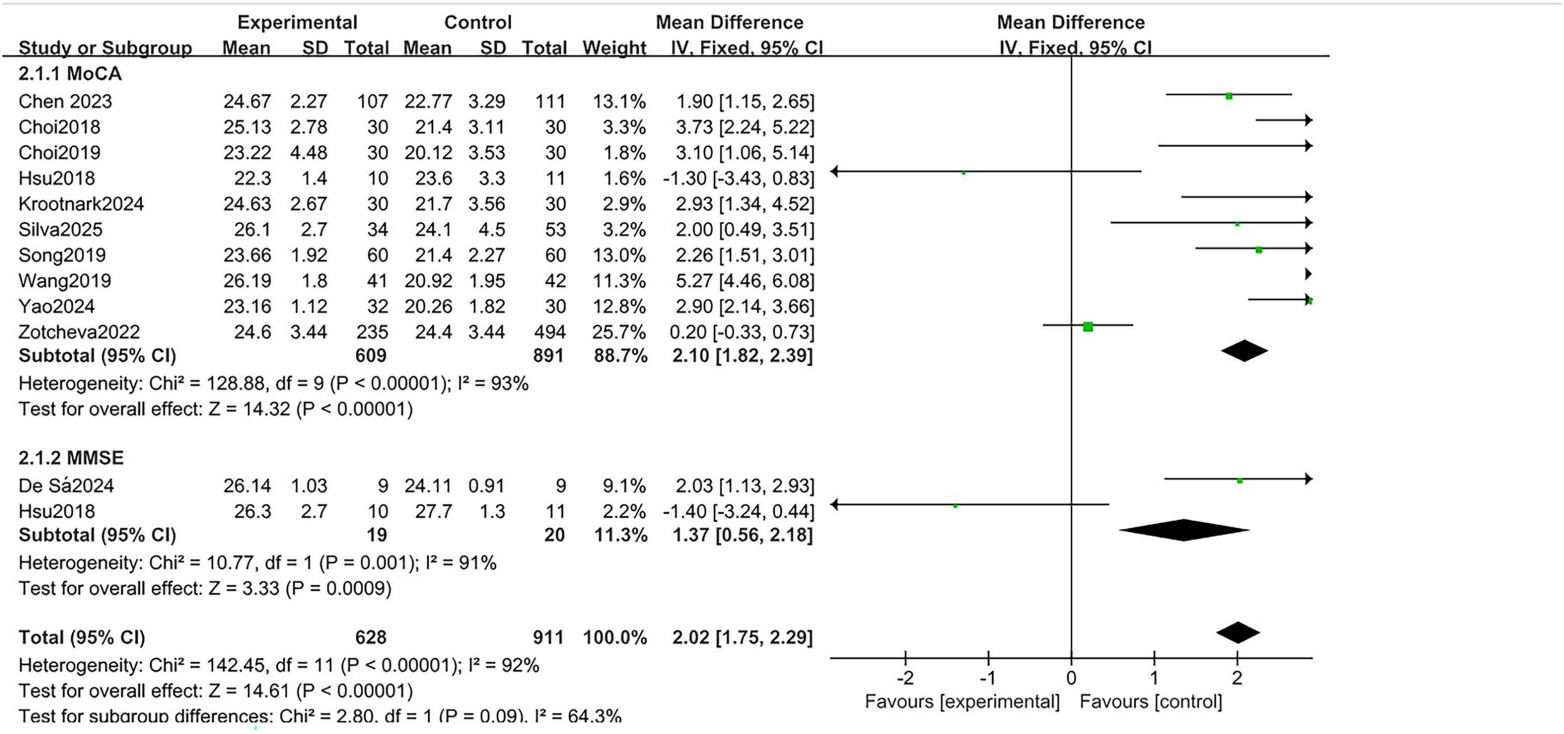
Aerobic exercise interventions on cognitive function in older adults with cognitive frailty.

The majority of studies (63%) met five or six of the seven quality criteria, with one meeting five and nine meeting all six, as shown in Supplementary Table S3. The research corpus was exclusively composed of randomized controlled trials (RCTs) with cognitive function as a primary endpoint. According to the quality appraisal, three studies met fewer than four items, resulting in a classification of very low quality. Owing to the pragmatic nature of the implemented interventions, the blinding of participants and research personnel was largely unattainable across the study cohort. The included studies, which sourced their samples from senior welfare centers, communities, hospitals, and clinics, were generally characterized by a low risk of bias. This methodological strength supports the broader relevance of the results. A comprehensive inclusion strategy was implemented, indicating that no study was excluded based on its quality assessment results. However, the included studies with significant heterogeneity were conducted in several countries, with different populations and cultures (Figure 2).

## 4 Discussion

To the best of our knowledge, this review represents the most comprehensive synthesis of aerobic exercise interventions for older adults with cognitive frailty. The majority of studies were published after 2013, reflecting the increasing prioritization of cognitive frailty in global aging and health agendas. Interventions, most commonly delivered face-to-face, ranged from walking and ground kayak paddling to enhanced Taichi, dual-task training, and aerobic dance, with durations of 3–6 months and session lengths of 45–90 min. Across studies, aerobic exercise yielded consistent improvements in global cognition, episodic memory, executive functions, balance, and mobility. Some trials further demonstrated biological signals compatible with neuroprotection, such as increased BDNF, reduced tau, or preserved hippocampal volume. Importantly, three studies that treated cognitive frailty as a secondary outcome still reported significant cognitive benefits, underscoring the value of integrating cognitive frailty endpoints into diverse exercise programs.

Aerobic exercise has been shown to enhance functional connectivity between the anterior putamen and sensorimotor cortex, improve cognitive control, increase connectivity in the right frontoparietal network in proportion to fitness gains, and reduce global brain atrophy (Johansson et al., 2022). Pure aerobic exercise likely improves cognition through systemic pathways: increased blood flow, higher BDNF levels, and reduced inflammation. Simultaneous dual-task and intrinsically embedded interventions provide additional cognitive benefits, requiring the brain to coordinate movement and attention simultaneously. This may engage prefrontal-parietal circuits more strongly. In contrast, sequential interventions deliver cognitive training after exercise.

Our findings confirmed and extended earlier meta-analyses demonstrating small-to-moderate cognitive benefits of aerobic exercise in older adults without dementia (Colcombe and Kramer, 2003; Northey et al., 2018) and in those with mild cognitive impairment (Baker et al., 2010). However, unlike previous reviews that focused on broad populations or single modalities, our synthesis directly compared four distinct aerobic exercise modalities: pure aerobic, sequential, simultaneous dual-task, and intrinsically embedded interventions (e.g., Taichi and Baduanjin).

This direct comparison represents a significant advancement over recent reviews. For example, a network meta-analysis by Peng et al. (2024) concluded that exercise benefits cognitive frailty, but it did not examine how cognitive tasks are integrated with physical activity (Peng et al., 2024). Another review by Liu et al. (2026) found that mind–body exercises (e.g., Baduanjin) improve cognition more than resistance training, but it did not analyze the timing of cognitive–physical integration (Liu et al., 2026). Our review fills these gaps by distinguishing motor complexity from explicit cognitive load.

This comparison revealed that aerobic exercise is not monolithic, and different modalities may confer distinct cognitive benefits. Enhanced Taichi outperformed both standard Taichi and fitness walking in global cognition. This suggests that embedding complex motor sequencing and attentional demands into aerobic movement may amplify neurocognitive gains. Similarly, simultaneous dual-task and cognitively enriched aerobic exercise yielded superior executive function improvements compared to low-cognitive-load aerobic exercise. This aligns with theories of simultaneous motor-cognitive training and enhances neural efficiency. Conversely, walking-based aerobic exercise still improved MoCA scores substantially compared to education controls. This further underscores its accessibility and scalability for older adults with low exercise capacity. In several head-to-head comparisons, aerobic exercise outperformed resistance training, stretching, or balance/toning programs in terms of global cognition, memory, and executive function. Aerobic exercise selectively promotes hippocampal volume and frontoparietal network integrity; this aligns with imaging evidence (Johansson et al., 2022; Erickson et al., 2011).

These findings have practical implications. For older adults with low fitness, simple walking-based aerobic exercise is a good first choice. It is effective, low-cost, and easy to implement in communities or at home. For those who can handle more complex activities, cognitively enriched modalities (dual-task walking, Taichi, Baduanjin, and adapted team sports) may provide extra benefits for executive function. Our results also suggest that sequential delivery (exercise then cognitive training) does not add value. Therefore, programs should integrate cognitive and physical tasks at the same time. Clinicians should also consider patient preferences. Team sports may improve adherence through social interaction. Home-based walking is more scalable in low-resource settings.

### 4.1 Temporal structure and cognitive components

The temporal relationship between physical and cognitive components is a critical modulator of neuroplastic effects. When cognitive tasks were embedded within or performed simultaneously with aerobic exercise, the temporal contiguity of these stimuli may have potentiated synaptic plasticity and facilitated more efficient neural adaptation. Conversely, sequential interventions where cognitive training occurred separately from exercise may have missed this synergistic window. This may explain why Silva et al. (2025) found no cognitive benefits despite improved physical function.

The superior outcomes observed in simultaneous dual-task interventions (Li et al., 2023; Suzuki et al., 2013) supported this temporal synergy hypothesis. By requiring participants to allocate attentional resources and coordinate cognitive-motor responses in real time, these protocols may have engaged prefrontal-hippocampal networks more intensively than sequential or exercise-only approaches. The sustained effects up to 48 weeks in cognitively enhanced Taichi (Li et al., 2023) further suggested that such temporally integrated training may induce lasting structural and functional brain changes.

For the cognitive frailty population, this temporal relationship may be particularly critical. Interventions that simultaneously target both domains may offer greater efficiency in reversing or delaying the mutually reinforcing cycle of cognitive and physical deterioration.

### 4.2 Exercise complexity vs. cognitive load

A key methodological consideration was the distinction between motor complexity and cognitive load. Motor complexity refers to the coordination demands inherent in learning skilled movements (e.g., Taichi forms and kayak paddling). Cognitive load denotes the explicit executive demands imposed by simultaneous dual-task protocols (e.g., walking while counting). These two factors often overlapped in practice, making it challenging to isolate the primary driver of cognitive improvement. The lack of standardized reporting on task ordering and cognitive loading further complicated interpretation. As such, whether superior outcomes were caused by motor complexity, cognitive load, or their interaction remains unclear. Future studies should systematically manipulate these components to disentangle their relative contributions.

### 4.3 Plausible mechanisms in the context of frailty

In the context of cognitive frailty, the mechanisms underlying aerobic exercise effects may involve shared pathways of inflammation and metabolic regulation (Angulo et al., 2020). Human studies have consistently shown that aerobic exercise increases peripheral levels of BDNF and Vascular Endothelial Growth Factor (VEGF), which correlate with cognitive improvement (Tsai et al., 2019). Recent animal research has further found that aerobic exercise enhances hippocampal BDNF expression and synaptic plasticity (Marcourt et al., 2025). Moreover, aerobic exercise mitigates systemic inflammation and insulin resistance, processes central to both cognitive decline and physical frailty (Kullmann et al., 2022; Gleeson et al., 2011).

### 4.4 Heterogeneity and implications

Heterogeneity in intervention type, intensity, and comparator likely contributed to variable effect sizes. Longer and more frequent programs generally produced larger cognitive gains; however, the evidence was not fully consistent. Even so, simple walking-based aerobic exercise improved MoCA scores significantly compared to education controls. This supports its use for older adults with low exercise capacity.

### 4.5 Limitations and future research directions

This review has several limitations. First, none of the included studies assessed physical frailty with validated tools. Therefore, our findings apply primarily to the cognitive dimension of cognitive frailty. Second, exercise intensity was reported inconsistently. Some studies used heart rate monitors. Others used subjective scales or gave no intensity information. This limits dose–response analysis. Third, only one sequential and two dual-task studies were available. Larger trials are needed to confirm the observed patterns. Fourth, the majority of studies had short follow-ups (≤6 months). Long-term effects on dementia risk remain unknown. Fifth, group-based interventions may have introduced social interaction as a confounder. Sixth, we limited our search to English and Chinese publications, which may introduce language bias. Seventh, several studies lacked pre-registered protocols. Future research should (1) include validated physical frailty measures; (2) standardize intensity reporting (e.g., using the Frequency; Intensity; Time;Type (FITT) principle); (3) extend follow-up to at least 12 months; (4) directly compare simultaneous vs. sequential designs within the same trial; and (5) explore how individual characteristics (age, sex, and baseline fitness) moderate responses to different modalities.

## Data Availability

The original contributions presented in the study are included in the article/Supplementary material, further inquiries can be directed to the corresponding author.

## References

[ref1] Angulo J. El Assar M. Álvarez-Bustos A. Rodríguez-Mañas L. (2020). Physical activity and exercise: strategies to manage frailty. Redox Biol. 35:101513. doi: 10.1016/j.redox.2020.101513, 32234291 PMC7284931

[ref2] Baker L. D. Frank L. L. Foster-Schubert K. Green P. S. Wilkinson C. W. McTiernan A. . (2010). Effects of aerobic exercise on mild cognitive impairment: a controlled trial. Arch. Neurol. 67, 71–79. 20065132 10.1001/archneurol.2009.307 PMC3056436

[ref3] Chen S. Chen T. Honda T. Kishimoto H. Nofuji Y. Narazaki K. . (2025). Cognitive frailty and functional disability in older adults: a 10-year prospective cohort study in Japan. Geroscience 47, 5057–5067. 39627573 10.1007/s11357-024-01461-0 PMC12181443

[ref4] Chen Y. Qin J. Tao L. Liu Z. Huang J. Liu W. . (2023). Effects of tai chi Chuan on cognitive function in adults 60 years or older with type 2 diabetes and mild cognitive impairment in China: a randomized clinical trial. JAMA Netw. Open 6:e237004. doi: 10.1001/jamanetworkopen.2023.7004, 37022680 PMC10080376

[ref5] Cheng M. Liu Q. Li M. He M. (2025). Cognitive frailty as a predictor of hospitalisation among older adults: a systematic review and meta-analysis. Psychogeriatrics 25:e13213. doi: 10.1111/psyg.13213, 39569746

[ref6] Cherbuin N. Northey J. M. Walsh E. I. Burns R. A. Speer H. Lawlis N. . (2026). A randomised controlled trial of physical activity and cognitive training in older adults: the PhABHeaD study. Trials. doi: 10.1186/s13063-026-09681-9, 42098784 PMC13321517

[ref7] Choi W. Lee S. (2018). Ground kayak paddling exercise improves postural balance, muscle performance, and cognitive function in older adults with mild cognitive impairment: a randomized controlled trial. Med. Sci. Monit. 24, 3909–3915. doi: 10.12659/MSM.908248, 29886507 PMC6026380

[ref8] Choi W. Lee S. (2019). The effects of virtual kayak paddling exercise on postural balance, muscle performance, and cognitive function in oder adults with mild cognitive impairment: a randomized controlled trial. J. Aging Phys. Act. 27, 861–870. doi: 10.1123/japa.2018-0020, 31034303

[ref9] Colcombe S. Kramer A. F. (2003). Fitness effects on the cognitive function of older adults: a meta-analytic study. Psychol. Sci. 14, 125–130. doi: 10.1111/1467-9280.t01-1-01430, 12661673

[ref10] De Sá C. A. Saretto C. B. Cardoso A. M. Remor A. Breda C. O. da Silva Corralo A. M. . (2024). Effects of a physical exercise or motor activity protocol on cognitive function, lipid profile, and BDNF levels in older adults with mild cognitive impairment. Mol. Cell. Biochem. 479, 499–509. doi: 10.1007/s11010-023-04733-z, 37186275

[ref11] Dhahbi W. Briki W. Heissel A. Schega L. Dergaa I. Guelmami N. . (2025). Physical activity to counter age-related cognitive decline: benefits of aerobic, resistance, and combined training-a narrative review. Sports Med Open 11:56. doi: 10.1186/s40798-025-00857-2, 40381170 PMC12085549

[ref12] Diniz B. S. Lima-Costa M. F. Peixoto S. V. Firmo J. O. A. Torres K. C. L. Martins-Filho O. A. . (2022). Cognitive frailty is associated with elevated proinflammatory markers and a higher risk of mortality. Am. J. Geriatr. Psychiatry 30, 825–833. doi: 10.1016/j.jagp.2022.01.012, 35227616 PMC9177532

[ref13] Dixe M. D. A. Pinho J. Pereira F. Verloo H. Meyer-Massetti C. Pereira S. G. . (2023). Patterns of medication management and associated medical and clinical features among home-dwelling older adults: a cross-sectional study in Central Portugal. Int. J. Environ. Res. Public Health 20:1701. doi: 10.3390/ijerph20031701, 36767067 PMC9914088

[ref14] Erickson K. I. Voss M. W. Prakash R. S. Basak C. Szabo A. Chaddock L. . (2011). Exercise training increases size of hippocampus and improves memory. Proc. Natl. Acad. Sci. USA 108, 3017–3022. doi: 10.1073/pnas.1015950108, 21282661 PMC3041121

[ref15] Facal D. Maseda A. Pereiro A. X. Gandoy-Crego M. Lorenzo-López L. Yanguas J. . (2019). Cognitive frailty: a conceptual systematic review and an operational proposal for future research. Maturitas 121, 48–56. doi: 10.1016/j.maturitas.2018.12.006 30704565

[ref16] Falbo S. Condello G. Capranica L. Forte R. Pesce C. . (2016). Effects of physical-cognitive dual task training on executive function and gait performance in older adults: a randomized controlled trial. Biomed. Res. Int. 2016:5812092. 28053985 10.1155/2016/5812092 PMC5178854

[ref17] Gates N. Fiatarone Singh M. A. Sachdev P. S. Valenzuela M. (2013). The effect of exercise training on cognitive function in older adults with mild cognitive impairment: a meta-analysis of randomized controlled trials. Am. J. Geriatr. Psychiatry 21, 1086–1097. doi: 10.1016/j.jagp.2013.02.018, 23831175

[ref18] Gleeson M. Bishop N. C. Stensel D. J. Lindley M. R. Mastana S. S. Nimmo M. A. (2011). The anti-inflammatory effects of exercise: mechanisms and implications for the prevention and treatment of disease. Nat. Rev. Immunol. 11, 607–615. doi: 10.1038/nri3041, 21818123

[ref19] Gong W. J. Zhang Y. M. (2023). Chinese expert consensus on cognitive frailty rehabilitation 2023. Chinese Journal of Medicine 58, 949–953.

[ref20] Hsu C. L. Best J. R. Davis J. C. Nagamatsu L. S. Wang S. Boyd L. A. . (2018). Aerobic exercise promotes executive functions and impacts functional neural activity among older adults with vascular cognitive impairment. Br. J. Sports Med. 52, 184–191. doi: 10.1136/bjsports-2016-096846, 28432077

[ref21] Huang Y. Ou H. Zhao W. Lin Q. Xue Y. Xia R. . (2024). The effects of moderate-intensity aerobic exercise on cognitive function in individuals with stroke-induced mild cognitive impairment: a randomized controlled pilot study. J. Rehabil. Med. 56:jrm33001. doi: 10.2340/jrm.v56.33001, 38956964 PMC11247515

[ref22] Huang J. H. Wang Q. S. Zhuo R. M. Su X. Y. Xu Q. Y. Jiang Y. H. . (2023). Institutional residence protects against cognitive frailty: a cross-sectional study. Inquiry 60:469580231220180. doi: 10.1177/00469580231220180, 38140825 PMC10748935

[ref23] Ibrahim N. A. Wong Y. Y. Lean Q. Y. Ramasamy K. Lim S. M. Tan M. P. . (2024). Medication self-management among older adults with cognitive frailty. Res. Social Adm. Pharm. 20, 172–181. doi: 10.1016/j.sapharm.2023.11.001 37980238

[ref24] Ichige M. H. A. Santos-Silva P. R. D'Andrea Greve J. M. (2026). Moderate and vigorous aerobic exercise enhances inhibitory control, but not working memory or cognitive flexibility, up to the second ventilatory threshold: a randomized crossover trial. Clinics (Sao Paulo) 81:100896. doi: 10.1016/j.clinsp.2026.100896, 41740527 PMC12973520

[ref25] Izquierdo M. Merchant R. A. Morley J. E. Anker S. D. Aprahamian I. Arai H. . (2021). International exercise recommendations in older adults (ICFSR): expert consensus guidelines. J. Nutr. Health Aging 25, 824–853. doi: 10.1007/s12603-021-1665-8, 34409961 PMC12369211

[ref26] Jenkins N. D. Welstead M. Stirland L. Hoogendijk E. O. Armstrong J. J. Robitaille A. . (2023). Frailty trajectories and associated factors in the years prior to death: evidence from 14 countries in the survey of health, aging and retirement in Europe. BMC Geriatr. 23:49. doi: 10.1186/s12877-023-03736-1, 36703138 PMC9881297

[ref27] Jia M. Hu F. Hui Y. Peng J. Wang W. Zhang J. (2025). Effects of exercise on older adults with mild cognitive impairment: a systematic review and network meta-analysis. J. Alzheimer's Dis 104, 980–994. doi: 10.1177/13872877251321176, 40026008

[ref28] Johansson M. E. Cameron I. G. M. Van der Kolk N. M. de Vries N. M. Klimars E. Toni I. . (2022). Aerobic exercise alters brain function and structure in Parkinson's disease: a randomized controlled trial. Ann. Neurol. 91, 203–216. doi: 10.1002/ana.26291, 34951063 PMC9306840

[ref29] Kim J. S. Kim E. (2024). Subjective memory complaints and medication adherence among hypertensive Korean older adults with multimorbidity: mediating effect of depression and social support. BMC Public Health 24:585. doi: 10.1186/s12889-024-18061-4, 38395841 PMC10885607

[ref30] Kocyigit S. E. Ates Bulut E. Aydin A. E. Dost F. S. Kaya D. Isik A. T. (2024). The relationship between cognitive frailty, physical frailty and malnutrition in Turkish older adults. Nutrition 126:112504. doi: 10.1016/j.nut.2024.112504, 39142070

[ref31] Krootnark K. Chaikeeree N. Saengsirisuwan V. Boonsinsukh R. (2024). Effects of low-intensity home-based exercise on cognition in older persons with mild cognitive impairment: a direct comparison of aerobic versus resistance exercises using a randomized controlled trial design. Front Med (Lausanne) 11:1392429. doi: 10.3389/fmed.2024.1392429 38975052 PMC11224483

[ref32] Kullmann S. Goj T. Veit R. Fritsche L. Wagner L. Schneeweiss P. . (2022). Exercise restores brain insulin sensitivity in sedentary adults who are overweight and obese. JCI Insight 7:e161498. doi: 10.1172/jci.insight.161498, 36134657 PMC9675563

[ref33] Li F. Harmer P. Eckstrom E. Fitzgerald K. Winters-Stone K. (2023). Clinical effectiveness of cognitively enhanced tai Ji Quan training on global cognition and dual-task performance during walking in older adults with mild cognitive impairment or self-reported memory concerns: a randomized controlled trial. Ann. Intern. Med. 176, 1498–1507. doi: 10.7326/M23-1603, 37903365 PMC13527859

[ref34] Li G. Teng G. Zhang W. Song T. Li Y. Wang Z. . (2025). Comparative effects of different physical exercises on cognitive function and intervention adherence in older adults with cognitive impairment: a systematic review and network meta-analysis. Clin. Psychol. Rev. 120:102604. doi: 10.1016/j.cpr.2025.102604 40466426

[ref35] Liu X. Cui C. Lv J. Zhang Y. (2026). The effects of traditional mind-body exercises on cognitive function in neurodegenerative diseases or prodromal cognitive decline: a meta-analysis. Front. Public Health 14:1735606. doi: 10.3389/fpubh.2026.1735606, 41756081 PMC12932201

[ref36] Liu J. Xu S. Wang J. Yan Z. Wang Z. Liang Q. . (2023). Prevalence of cognitive frailty among older adults in China: a systematic review and meta-analysis. BMJ Open 13:e066630. doi: 10.1136/bmjopen-2022-066630, 37076151 PMC10124291

[ref37] Makino T. Umegaki H. Ando M. Cheng X. W. Ishida K. Akima H. . (2021). Effects of aerobic, resistance, or combined exercise training among older adults with subjective memory complaints: a randomized controlled trial. J. Alzheimer's Dis 82, 701–717. doi: 10.3233/JAD-210047 34092635

[ref38] Marcourt C. Pin-Barre C. Langeard A. Rivera C. Temprado J. J. Laurin J. (2025). Cognitive and sensorimotor benefits of moderate- and high-intensity exercise are associated with specific expression of neurotrophic markers in older rats. Sci. Rep. 15:6292. doi: 10.1038/s41598-025-90719-4, 39984706 PMC11845600

[ref39] Northey J. M. Cherbuin N. Pumpa K. L. . (2018). Exercise interventions for cognitive function in adults older than 50: a systematic review with meta-analysis. Br. J. Sports Med. 52, 154–160. doi: 10.1136/bjsports-2016-096587, 28438770

[ref40] Park J. H. (2022). Effects of acute moderate-intensity aerobic exercise on executive function and prefrontal cortex activity in community-dwelling older adults: a single-blind, randomized controlled trial. Geriatr Gerontol Int 22, 227–232. doi: 10.1111/ggi.14352, 35083837

[ref41] Peng J. Chang R. Wei X. Yin Z. Liu Q. (2024). Effect of non-pharmacological interventions in people with cognitive frailty: a systematic review and network meta-analysis. BMC Public Health 24:2684. doi: 10.1186/s12889-024-20079-7, 39354435 PMC11443714

[ref42] Silva A. F. Clemente F. M. Roriz M. S. Azevedo J. A. Jovanovic O. Adamovic M. . (2025). The effect of aerobic or strength training in elderly with cognitive decline: the Fit4Alz project. J. Sports Sci. Med. 24, 172–186. doi: 10.52082/jssm.2025.172, 40046223 PMC11877294

[ref43] Song D. Yu D. S. F. (2019). Effects of a moderate-intensity aerobic exercise programme on the cognitive function and quality of life of community-dwelling elderly people with mild cognitive impairment: a randomised controlled trial. Int. J. Nurs. Stud. 93, 97–105. doi: 10.1016/j.ijnurstu.2019.02.019, 30901716

[ref44] Suzuki T. Shimada H. Makizako H. Doi T. Yoshida D. Ito K. . (2013). A randomized controlled trial of multicomponent exercise in older adults with mild cognitive impairment. PLoS One 8:e61483. doi: 10.1371/journal.pone.0061483, 23585901 PMC3621765

[ref45] Talar K. Vetrovsky T. van Haren M. Négyesi J. Granacher U. Váczi M. . (2022). The effects of aerobic exercise and transcranial direct current stimulation on cognitive function in older adults with and without cognitive impairment: a systematic review and meta-analysis. Ageing Res. Rev. 81:101738. doi: 10.1016/j.arr.2022.101738 36162707

[ref46] Tari A. R. Walker T. L. Huuha A. M. Sando S. B. Wisloff U. (2025). Neuroprotective mechanisms of exercise and the importance of fitness for healthy brain ageing. Lancet 405, 1093–1118. doi: 10.1016/S0140-6736(25)00184-9, 40157803

[ref47] ten Brinke L. F. Bolandzadeh N. Nagamatsu L. S. Hsu C. L. Davis J. C. Miran-Khan K. . (2015). Aerobic exercise increases hippocampal volume in older women with probable mild cognitive impairment: a 6-month randomised controlled trial. Br. J. Sports Med. 49, 248–254. doi: 10.1136/bjsports-2013-093184, 24711660 PMC4508129

[ref48] Thompson P. D. Arena R. Riebe D. Pescatello L. S. . American College of Sports Medicine. (2013). ACSM’S new preparticipation health screening recommendations from ACSM’S guidelines for exercise testing and prescription, ninth edition. Curr. Sports Med. Rep. 12, 215–217. doi: 10.1249/JSR.0b013e31829a68cf, 23851406

[ref49] Trombini-Souza F. de Moura V. T. G. da Silva L. W. N. Leal I. S. Nascimento C. A. Silva P. S. T. . (2023). Effects of two different dual-task training protocols on gait, balance, and cognitive function in community-dwelling older adults: a 24-week randomized controlled trial. PeerJ 11:e15030. doi: 10.7717/peerj.15030, 37101796 PMC10124542

[ref50] Tsai C. L. Pai M. C. Ukropec J. Ukropcová B. (2019). Distinctive effects of aerobic and resistance exercise modes on neurocognitive and biochemical changes in individuals with mild cognitive impairment. Curr. Alzheimer Res. 16, 316–332. doi: 10.2174/1567205016666190228125429 30819077

[ref51] Varrasi S. Castellano S. Magistro D. (2024). Editorial: the open challenges of cognitive frailty: risk factors, neuropsychological profiles and psychometric assessment for healthy aging. Front. Aging Neurosci. 17:1679406. 10.3389/fnagi.2025.1679406 PMC12401963 40904346

[ref52] Wang Y. W. Li X. D. Bi X. J. Zhao C. S. . (2019). Effects of aerobic exercise on cognitive function in elderly community-dwelling adults with mild cognitive impairment. Chin. J. Gerontol. 39, 848–850.

[ref53] Wang Z. Lin L. Zuo S. Ye C. Huang X. Xu Y. (2025). Prevalence of cognitive frailty among Chinese older adults: a systematic review and meta-analysis. Gen. Hosp. Psychiatry 96, 156–167. doi: 10.1016/j.genhosppsych.2025.07.008, 40674778

[ref54] Xia J. Zou Y. Cui Y. Zhang S. Huo K. Liu W. . (2025). Physical exercise activates a PVN-NAc oxytocin circuit to relieve stress-induced depressive-like behaviors. Proc. Natl. Acad. Sci. USA 122:e2503675122. doi: 10.1073/pnas.2503675122, 40392854 PMC12130824

[ref55] Xu W. L. Qian C. Xie Y. . (2023). Effects of baduanjin exercise on memory in elderly patients with amnestic mild cognitive impairment. Modern J Integr Tradit Chin Western Med 32, 1260–1263, +1268.

[ref56] Yao L. Yang Q. Hu X. . (2024). Delaying effect of Baduanjin exercise combined with cup-stacking training on the progression of amnestic mild cognitive aging in the elderly. Chin. J. Gerontol. 44, 3904–3907.

[ref57] You H. S. Kwon Y. J. Kim S. Kim Y. H. Kim Y. S. Kim Y. . (2021). Clinical practice guidelines for managing frailty in community-dwelling Korean elderly adults in primary care settings. Korean J Fam Med 42, 413–424. doi: 10.4082/kjfm.21.0162, 34871482 PMC8648485

[ref58] Yu X. Qian Y. Zhang Y. Chen Y. Wang M. (2024). Association between polypharmacy and cognitive impairment in older adults: a systematic review and meta-analysis. Geriatr. Nurs. 59, 330–337. doi: 10.1016/j.gerinurse.2024.07.005, 39111065

[ref59] Yuan Y. Wang S. Zhou C. Zhang A. Zhang S. Wang Y. (2025). Effects of exercise interventions on cognition, physical function and quality of life among older adults with cognitive frailty: a systematic review and meta-analysis. Geriatr. Nurs. 62, 96–107. doi: 10.1016/j.gerinurse.2025.01.006, 39889512

[ref60] Zang W. Fang M. Xiao N. Wu J. Zhang Q. Mao X. (2026). Exercise prescriptions for older adults with different degrees of cognitive impairment: a dose-response network meta-analysis. Clin. Rehabil. 40, 154–170. doi: 10.1177/02692155251385219, 41556956

[ref61] Zhang Y. Xia H. Jiang X. Wang Q. Hou L. (2024). Prevalence and outcomes of cognitive frailty among community-dwelling older adults: a systematic review and Meta-analysis. Res. Gerontol. Nurs. 17, 202–212. doi: 10.3928/19404921-20240621-01 39047228

[ref62] Zotcheva E. Håberg A. K. Wisløff U. Salvesen Ø. Selbæk G. Stensvold D. . (2022). Effects of 5 years aerobic exercise on cognition in older adults: the generation 100 study: a randomized controlled trial. Sports Med. 52, 1689–1699. doi: 10.1007/s40279-021-01608-5, 34878637 PMC9213353

