# Supplementary material for: Aerobic exercise interventions for older adults with cognitive frailty: a systematic review
Source: Front Aging Neurosci. 2026 Jun 25;18:1747309. doi: 10.3389/fnagi.2026.1747309 (PMC13345865; doi:10.3389/fnagi.2026.1747309)
Supplement: Supplementary file 1 [file Data_Sheet_1.DOCX]

Table S1 intervention

| **Author, year, country** | **Type of intervention** | **Mode of intervention** | **Intervention content** | **Intervention frequency** | **Duration of intervention** | **Duration per session** | **Intervention provider** | **Intervention adherence or acceptance rate** | **Theroretical basis of intervention** | **Intervention evaluation time points** | **exercise modality** |
| --- | --- | --- | --- | --- | --- | --- | --- | --- | --- | --- | --- |
| Choi et al., 2018, South Korea [37] | Behavioral | Face to face | IG: Ground Kayak Paddling Exercise  CG: home exercise program | Twice a week | 6 weeks | 60 -minute | One instructor led the program and 2 assistants | 96% | Aerobic exercise reduces the loss of brain tissue in older adults | Baseline  8 weeks | Intrinsic / Embedded interventions |
| Krootnark et al., 2024, Thailand [38] | Behavioral | Face to face + telephone | The aerobic exercise group:  A home-based aerobic exercise program The resistance exercise  group:  A home-based resistance training program  CG: Continue their usual daily life activitie | 35min/day, 5days/week | 3months | Starting 15 min per day and increasing by 5 min every 2 weeks. | Exercise program instructor | 94.83% | Aerobic and resistance exercise have impact on cognitive performance in older person with MCI | Baseline  3 months  6 months | Pure aerobic exercise |
| Suzuki et al.,  2013  Japan [39] | Behavioral | Face to face | IG: Aerobic exercise, muscle strength training, postural balance retraining, and dual-task training | Biweekly | 6months | 90-minute | Physiotherapist | 94% | Physical activity is associated with improvements in attention, processing speed, and executive function in older adults with and without cognitive impairments | Baseline  6 months | Simultaneous / Dual‑Task interventions |
| Ten Brinke et al., 2015，Netherlands [40] | Behavioral | Face to face | RT: Mini-squats, Mini-lunges and lunge walks.  AT: An outdoor walking programme  CG: Balance and tone training | Twice-weekly | 6 months | 60-minute | Instructors | 60% | Exercise is a promising strategy for combating cognitive decline. | Baseline  6 months | Pure aerobic exercise |
| Zotcheva et al.,2022, Norway [41] | Behavioral | Face to face | MICT: Moderate-intensity continuous training  HIIT: High-intensity continuous training  CG: Recommended  to follow national recommendations for physical activity | Twice weekly | 5 years | 50-minute | Instructors | 76% | Longer exercise interventions appear to have a greater impact on cognition | Baseline  1 year  3 years  5 years | Pure aerobic exercise |
| Wang et al., 2019,China [42] | Behavioral | Face to face | IG: gymnastic exercises + community usual health education  CG: community usual health education | More than 5 times per week | 3 months | More than 45-minute | Physical education teacher, epidemiology teacher, etc. | 95.34% | Gymnastics is a low-to-moderate intensity aerobic exercise. By increasing blood flow to brain tissue and stimulating the excitability of the central nervous system, it improves cognitive function in older adults. | Baseline  3 months | Pure aerobic exercise |
| Xu et al.,2023, China [43] | Behavioral | Face to face | IG:  Routine care and health education + Baduanjin  CG: Routine care and health education | 3 times per week | 12 weeks | 50-minute | Doctor | 91.43% | Ba Duan Jin (Eight-Section Brocade) can unblock the meridian channels in the human body and improve cognitive function | Baseline  12 weeks | Intrinsic / Embedded interventions |
| Yao et al., 2024, China [44] | Behavioral | Face to face | IG:  Routine care and health education + Baduanjin  CG: Routine care and health education | 5 times per week | 6 months | 45-minute | Doctor | 100% | / | Baseline  6 months | Intrinsic / Embedded interventions |
| Choi et al., 2019, Republic of Korea [45] | Behavioral | Face to face | IG: Kayaking group exercise  CG: Home exercise | Twice a week | 6 weeks, | 60-minute | Instructors | 95% | / | Baseline  6 weeks | Intrinsic / Embedded interventions |
| De Sá et al.,2024, Brazil [46] | Behavioral | Face to face | PE: Physical exercise  MT: The motor task complexity protocol  PE+MT: Physical exercise +the motor task complexity protocol | Twice a week | 6 months | 60-minute | Researchers | 58.7% | Two-dimensional model of Gentile's taxonomy | Baseline  6 months | Intrinsic / Embedded interventions |
| Hsu et al.,2018, Canada [47] | Behavioral | Face to face | IG: Aerobic training  CG: Usual care | Thrice-weekly | 6 months | 60-minute | Researchers | 30% | Targeted aerobic training improves vascular function and alters inflammatory response during ischaemia. | Baseline  6 months | Pure aerobic exercise |
| Li et al., 2023, USA [48] | Behavioral | Online | Enhanced tai ji quan: 8 tai ji quan forms + practice in dynamic tai ji quan forms  Standard tai ji quan: practice of 8 tai ji quan forms Stretching exercise:  stretching exercise | twice a week | 24 weeks | 60-minute | researchers | 97% | Tai ji quan, a moderate-intensity mind–body exercise, improves cognition in healthy older adults | Baseline  16 weeks  24 weeks  48 weeks | Simultaneous / Dual‑Task interventions |
| Makino et al.,2021,Japan [49] | Behavioral | Face to face | AT:  Step-in-place exercises + A walking program  RT:  Elastic resistance training  + Bodyweight exercises  CT: combined the AT and RT programs  CG: attend educational classes | Twice a week | 26 weeks | 60-minute | Instructor | 91% | AT or RT have been the main exercise interventions and each has 76 been associated with cognitive improvement | Baseline  26 weeks  52 weeks | Pure aerobic exercise |
| Silva et al.,2025, Portugal [50] | Behavioral | Face to face | STCT: strength plus cognitive training  ST: strength training  AT: aerobic training  ATCT: aerobic plus cognitive training | 3 time a week | 12 weeks | 60-minute | Instructor | 80.20% | The cognitive benefits of PE is its ability to enhance blood flow and vascularization, leading to improved oxygen and nutrient delivery to the brain | Baseline  12 weeks | Sequential interventions |
| Song et al.,  2019,China [51] | Behavioral | Face to face | IG: Aerobic stepping exercise programme  CG: Health education programme | Once a week | 16 weeks | 60-minute | Nurse | 79.2% | The cognitive benefits of different exercise modalities amongst the individuals with mild cognitive impairment have been reported. | Baseline  16weeks | Pure aerobic exercise |
| Chen et al.,2023, China [52] | Behavioral | Face to face | Tai chi chuan group: Tai chi chuan+ education  Fitness walking group: Fitness walking training+ education  CG: Self-management education | 3 time a week | 24 weeks | 60-minute | Instructors | 88.1% | Tai chi chuan incorporates physical, cognitive, social. | Baseline  24 weeks  36 weeks | Intrinsic / Embedded interventions |

Table S2 Presents information on the direction of effects in the included studies

| Studies | Scale | Score | |
| --- | --- | --- | --- |
|  |  | Within group | Between group |
| Choi et al., 2018, South Korea [37] | MocA | Baseline 21.66±3.24; 8 weeks: 25.13 ±2.78^d^ | 8 weeks: IG:25.13 ±2.78; CG: 21.4±3.11^d^ |
| Krootnark et al., 2024, Thailand [38] | **MoCA** | The aerobic exercise group:  Baseline 20.17±2.09; 3months: 25.40±2.54^b^  6 months: 24.63±2.67^b^ | The aerobic exercise group:  3months: 25.40±2.54; 6 months:24.63±2.67  The resistance exercise group:  3months: 24.53±2.90; 6 months: 23.47±3.21  CG:  3months:21.17±2.77^b^; 6 months: 21.7±3.56^b^ |
|  | **TMT-A (Processing speed)** | The aerobic exercise group:  Baseline 60.01±17.41; 3month: 39.53±10.70^b^;  6 months: 47.75±16.42^b^ | The aerobic exercise group:  3month: 39.53±10.70; 6 months:47.75±16.42  The resistance exercise group:  3month: 45.83±22.92; 6 months:50.91±27.92  CG:  3month: 65.68±36.37^b^; 6 months:64.96±31.78^b^ |
|  | **TMT-B (Mental flexibility)** | The aerobic exercise group:  Baseline 174.95±174.26; 3months:78.26± 34.08^b^;  6 months:88.55±44.77^b^ | The aerobic exercise group:  3months:78.26± 34.08; 6 months:88.55±44.77  The resistance exercise group:  3months: 88.26±52.83; 6 months:103.22±68.97  CG:3months: 154.36±92.64^b^;  6 months: 160.48±115.08^b^ |
|  | **SCWT (Inhibitory control)** | The aerobic exercise group:  Baseline -13.66±6.43; 3 months: -0.38±6.78^b^;  6 months:-5.14±8.85^b^ | The aerobic exercise group:  3months: -0.38±6.78; 6 months: -5.14±8.85  The resistance exercise group:  3months:-4.64±6.79^b^; 6 months: -8.62±7.75  CG:  3months: -12.25±6.46^b^; 6 months:-11.48± 6.13^b^ |
|  | **DST-F (Short-term memory)** | The aerobic exercise group:  Baseline 6.27±1.34; 3months: 7.90±1.27^b^;  6 months: 7.07±1.20^b^ | The aerobic exercise group:  3months: 7.90±1.27; 6 months:7.07±1.20  The resistance exercise group:  3months: 7.50±1.20; 6 months: 6.70±1.32  CG: 3months:6.53±1.11^b^; 6 months: 6.60±0.93 |
|  | **DST-B (Working memory)** | The aerobic exercise group:  Baseline 2.90±0.61; 3months: 3.67±1.09^b^;  6 months:3.47±1.14^b^ | The aerobic exercise group:  3months: 3.67±1.09; 6 months:3.47±1.14  The resistance exercise group:  3month: 3.40±0.77; follow-up:3.17±0.59  CG: 3month: 3.00±0.59^b^; follow-up: 3.07±0.52 |
|  | **SDT ( Visuoconstructional reasoning)** | The aerobic exercise group:  Baseline 9.27±1.53; 3months:9.67±1.49;  6 months: 9.47±1.17 | The aerobic exercise group:  3months:9.67±1.49; 6 months: 9.47±1.17  The resistance exercise group:  3months: 9.77±1.63; 6 months:9.50±1.31  CG: 3month: 9.47±1.41; 6 months:9.33±1.40 |
|  | **TUG-M (Physical mobility)** | The aerobic exercise group:  Baseline 14.56±3.02; 3 months:11.24±1.79^b^;  6 months: 13.03±3.73^b^ | The aerobic exercise group:  3months:11.24±1.79; 6 months: 13.03±3.73  The resistance exercise group:  3months: 11.94±3.07; 6 months:13.66±3.73  CG: 3month: 15.38±5.55^b^; follow-up:15.26±5.79 |
| Suzuki et al.,2013,Japan [39] | **MMSE** | IG  Baseline 26.80±2.30; 6months: 0.20[-0.5,0.9]^b^ | IG: 6months: 0.20[-0.5,0.9]  CG: 6months: -0.30[-1.1,0.4]^b^ |
|  | **ADAS-cog** | IG  Baseline 6.00±2.80; 6months: -0.8[-1.4,-0.2]^c^ | IG: 6months: -0.8[-1.4,-0.2]  CG: 6months: -0.2[-0.8,0.4]^b^ |
|  | **WMS-LM I (Immediate recall)** | IG  Baseline 14.60±6.90; 6months: 2.8[1.4,4.2]^d^ | IG: 6months: 2.8[1.4,4.2]  CG: 6months: 1.0[-0.5,2.4]^d^ |
|  | **WMS-LM II(Delayed recall)** | IG  Baseline 10.50±7.40; 6months: 3.4[2.0,4.8]^d^ | IG: 6months: 3.4[2.0,4.8]  CG: 6months: 1.9[0.4,3.4]^d^ |
|  | **MTA-ERC(medial temporal areas including the entorhinal cortex)** | IG  Baseline 1.30±0.90; 6months: 0[-0,0.1] | IG: 6months: 0[-0,0.1]  CG: 6months: 0[0,0.1] |
|  | **WBC atrophy level** | IG  Baseline 7.30±4.70; 6months: 0.1[-0.4,0.7]^b^ | IG: 6months: 0.1[-0.4,0.7];  CG: 6months: 0.7[0.1,1.2]^b^ |
| Ten Brinke et al., 2015，Netherlands [40] | **Hippocampus volume baseline (mm3)**  **Total hippocampus** | AT:  Baseline:6495.30±1072.08;6months: 6756.20±952.25 | AT:6months: 6756.20±952.25;  BAT:6months: 5951.36±1117.94^c^ |
|  | **Hippocampus volume baseline (mm3)**  **Left hippocampus** | AT:  Baseline:3158.20±500.41.08;  6months: 3336.50±454.99 | AT:  6months: 3336.50±454.99  BAT:  6months: 3027.73±563.30^b^ |
|  | **Hippocampus volume baseline (mm3)**  **Right hippocampus** | AT:  Baseline:3337.10±656.34; 6months: 3419.70±539.57 | AT: 6months: 3419.70±539.57  BAT: 6months: 2923.64±626.61^b^ |
| Zotcheva et al.,2022, Norway [41] | **MoCA** | / | MICT: 5 years 24.60±3.44  HIIT: 5 years 24.70±3.11  CG: 5 years 24.40±3.44 |
| Wang et al., 2019,China [42] | **MoCA** | IG: Baseline 20.35±2.99; 3month: 26.19±1.80^b^ | IG: 3month 26.19±1.80;CG: 3month 20. 92±1.95^b^ |
| Xu et al.,2023, China [43] | **MoCA** | IG: Baseline21[20-24]; 3month: 24[22,25]^b^ | IG: 24[22,25]; CG: 22[19.25-23]^b^ |
|  | **AVLT-H**  **N1 immediate memory** | IG: Baseline 4[2,5]; 3month: 4[3,6]^b^ | IG: 4[3,6]; CG: 3[2,4]^b^ |
|  | **N2 immediate memory** | IG: Baseline 5[3,7]; 3month: 5[4,8]^b^ | IG: 5[4,8]; CG: 4[3,5.75]^b^ |
|  | **N3 immediate memory** | IG: Baseline 6[4,8]; 3month: 7[4,8]^b^ | IG: 7[4,8]; CG: 5[4.25,7]^b^ |
|  | **N4** **Short-term delayed recall** | IG: Baseline 5[3,7]; 3month: 6[5,8]^b^ | IG: 6[5,8]: CG: 4.5[4,6]^b^ |
|  | **N5 Long-term delayed recall** | IG: Baseline 6[3,8]; 3month: 7[4,9]^b^ | IG: 7[4,9]; CG: 5[4,6]^b^ |
|  | **N6 Cued recall** | IG:Baseline 4[2,6]; 3month: 5[3,8]^b^ | IG: 5[3,8]; CG:4[2.25,4.75]^b^ |
|  | **N7 Recognition** | IG: Baseline 9[6,11]; 3month: 10[7,12]^b^ | IG: 10[7,12]; CG: 9[7.2,10]^b^ |
| Yao et al., 2024, China [44] | **MoCA** | IG: Baseline20. 71±2. 23; 6month: 23. 16±1. 12^b^ | IG: 23. 16±1. 12; CG: 20. 26±1. 82^b^ |
|  | **ADCS-ADL** | IG: Baseline57.98±5. 23; 6month: 62.02±6.28^b^ | IG: 62.02±6.28; CG: 57.66±5.12 ^b^ |
|  | **PSQI** | IG: Baseline 4[4,5]; 6month: 4[4,5]^b^ | IG: 4[4,5]; CG:4[4,5]^b^ |
| Choi et al., 2019, Republic of Korea [45] | **Eyes open MLS (mm/s)** | IG: Baseline4.00 ± 1.59 ;6 weeks: 3.37 ± 0.91^b^ | IG: 3.37 ± 0.91; CG 3.74 ± 1.10^a^ |
|  | **Static balance APS (mm/s)** | IG: Baseline 5.87 ± 1.80; 6 weeks: 5.12 ± 1.27^b^ | IG: 5.12 ± 1.27; CG: 5.39 ± 1.06^b^ |
|  | **Static balance VM (mm2/s)** | IG:Baseline5.10 ± 3.70 ;6weeks:3.65 ± 1.91^b^ | IG: 3.65 ± 1.91; CG: 5.43 ± 3.21^b^ |
|  | **Eyes closed MLS (mm/s)** | IG: Baseline2.97 ± 1.28;6weeks: 2.62 ± 0.98^b^ | IG: 2.62 ± 0.98; CG: 2.90 ± 0.71^b^ |
|  | **Eyes closed APS (mm/s)** | IG: Baseline 4.68 ± 1.23;6weeks: 4.36 ± 0.86^b^ | IG: B4.36 ± 0.86 ; CG: 4.62 ± 1.07^b^ |
|  | **Eyes closed VM (mm2/s)** | IG: Baseline 2.98 ± 4.22 ;6weeks:2.34 ± 3.59^b^ | IG: 2.34 ± 3.59 ; CG: 3.16 ± 1.37^b^ |
|  | **Right OLS (s)** | IG: Baseline 10.41 ± 13.08 6weeks:21.76 ± 30.25^b^ | IG: 21.76 ± 30.25 ; CG: 11.32 ± 7.63^b^ |
|  | **Left OLS (s)** | IG: Baseline 11.12 ± 14.71;6weeks: 22.99 ± 31.05^b^ | IG: 22.99 ± 31.05 ; CG: 12.93 ± 10.70^b^ |
|  | **Dynamic balance TUG (s)** | IG: Baseline 10.21 ± 1.93 6weeks:8.86 ± 1.85^b^ | IG: b8.86 ± 1.85 ; CG: b9.97 ± 1.32^b^ |
|  | **Dynamic balance FRT (cm)** | IG: Baseline 26.14 ± 6.67 ;6 weeks:30.46 ± 5.86^b^ | IG: 30.46 ± 5.86 ; CG: 29.82 ± 6.39^b^ |
|  | **Dynamic balance BBS (point)** | IG: Baseline 50.13 ± 4.75;6weeks: 54.24 ± 1.99^b^ | IG: 54.24 ± 1.99 ; CG: 50.78 ± 2.41^b^ |
|  | **Dynamic balance FSST (s)** | IG: Baseline 9.55 ± 2.04; 6weeks: 8.02 ± 1.57^b^ | IG: 8.02 ± 1.57; CG: 9.08 ± 2.15^b^ |
|  | **Cognitive function MoCA** | IG: Baseline 21.10 ± 4.93; 6weeks: 23.22±4.48^b^ | IG: 23.22±4.48; CG: 20.12 ± 3.53^a^ |
|  | **Cognitive function GPCOG(point)** | IG: Baseline 10.56± 3.08; 6weeks: 11.98±2.26^b^ | IG: 11.98±2.26; CG: 10.97± 2.22^b^ |
| De Sá et al.,2024, Brazil [46] | **MMSE** | PE+MT: Baseline24.14±1.06; 6months:26.14±1.03^b^  PE: Baseline22.56±0.94; 6months:24.11±0.19^b^ | PE: 24.11±0.91; MT: 24.89±0.91;  PE+MT: 26.14±1.03 |
| Hsu et al.,2018, Canada [47] | **MMSE** | IG: baseline26.80±2.30; 6months:26.30±2.70 | IG: 26.30±2.70; CG: 27.7±1.3 |
|  | **MoCA** | IG: baseline22.2±2.4; 6months:22.3±1.4 | IG: 22.3±1.4; CG：23.6±3.3 |
| Li et al., 2023, USA [48] | **The change of MoCA score** | Cognitively enhanced Tai Ji: 3.1[2.6,3.7] | Cognitively enhanced Tai Ji: 3.1[2.6,3.7]  Standard Tai Ji Quan: 1.7[1.1,2.3]  Stretching exercise: 0.3[-0.2,0.8] |
|  | **The reduction of dual-task costs** | Cognitively enhanced Tai Ji: 21%;  Standard Tai Ji Quan: 11% | Cognitively enhanced Tai Ji: 21%;  Standard Tai Ji Quan: 11%  Stretching exercise: -1.2% |
|  | **Differences change of CDR–Sum of Boxes score** | Cognitively enhanced Tai Ji:  16 weeks: 0.50±0.10 ; 24 weeks: 0.30 ±0.10 | Cognitively enhanced Tai Ji:  16weeks: 0.50±0.10 ; 24 weeks: 0.30 ±0.10  Standard Tai Ji Quan:  16 weeks: 0.60±0.05; 24 weeks: 0.60 ±0.05  Stretching exercise  16 weeks: 0.90±0.10 ; 24 weeks: 0.90±0.10 |
|  | **Differences change of Trail Making Test-B, s** | Cognitively enhanced Tai Ji:  16 weeks: 80.6± 1.70; 24 weeks: 72.40±2.00 | Cognitively enhanced Tai Ji:  16 weeks: 80.6± 1.70; 24 weeks: 72.40±2.00  Standard Tai Ji Quan:  16 weeks: 88.3±1.60; 24 weeks: 86.20±2.00±  Stretching exercise  16 weeks: 95.20 ±1.70 ; 24 weeks: 94.20±2.10 |
|  | **Differences change of Forward Digit Span score** | Cognitively enhanced Tai Ji:  16 weeks: 11.90 ±0.20 ; 24 weeks: 12.80±0.20 | Cognitively enhanced Tai Ji:  16 weeks: 11.90 ±0.20 ; 24 weeks: 12.80±0.20  Standard Tai Ji Quan:  16 weeks: 11.70±0.20 ; 24 weeks: 12.30±0.20  Stretching exercise  16 weeks: 10.90± 0.20; 24 weeks: 10.70±0.20 |
|  | **Differences change of Backward Digit Span** | Cognitively enhanced Tai Ji:  16 weeks: 9.30±0.20 ; 24 weeks: 10.40±0.20 | Cognitively enhanced Tai Ji:  16 weeks: 9.30±0.20 ; 24 weeks: 10.40±0.20  Standard Tai Ji Quan:  16 weeks: 9.0±0.20; 24 weeks: 9.40±0.20  Stretching exercise  Baseline: 8.40±0.20; 16 weeks: 8.40±0.20 ;  24 weeks: 8.30±0.20 |
|  | **Differences change of Verbal fluency** | Cognitively enhanced Tai Ji:  16 weeks: 18.50 ±0.30 ; 24 weeks: 19.70±0.40 | Cognitively enhanced Tai Ji:  16 weeks: 18.50 ±0.30 ; 24 weeks: 19.70±0.40  Standard Tai Ji Quan:  16 weeks: 17.70±0.30; 24 weeks: 18.8±0.40  Stretching Exercise  16 weeks: 15.00±0.30 ; 24 weeks: 15.40±0.40 |
| Makino et al.,2021,Japan [49] | **MMSE** | AT: 0.99[0.52,1.46] | AT: 0.99[0.52,1.46]; RT: 0.43[-0.05,-0.89]  CT: 0.82[0.37,1.27]; CG: 0.45[-0.02-0.92]^a^ |
|  | **WMS-R Logical Memory I** | AT: 2.68[1.58,3.78] | AT: 2.68[1.58,3.78]; RT: 3.15[2.05,4.25]  CT: 3.14[2.10,4.18]; CG: 2.98[1.88,4.08]^b^ |
|  | **WMS-R Visual Reproduction I** | AT: 1.90[1.04,2.76] | AT: 1.90[1.04,2.76]; RT: 1.02[0.16,1.88]  CT: 1.56[0.74,2.38]; CG: 1.32[0.46,2.18] |
|  | **WMS-R Logical Memory II** | AT:3.70[2.62,4.78] | AT:3.70[2.62,4.78]; RT: 4.03[2.95,4.03]  CT:3.87[2.83,4.9]; CG: 3.45[2.35,4.55]^b^ |
|  | **WMS-R Visual Reproduction II** | AT: 4.73[3.34,6.12] | AT: 4.73[3.34,6.12]; RT: 4.47[3.08,5.86]  CT: 4.32[2.97,5.67]; CG: 5.56[4.15,6.97] |
|  | **WMS-R Digit Span Forward** | AT: -0.45[-0.47,-0.16] | AT: -0.45[-0.47,-0.16]; RT: 0.13[-0.16,0.42]  CT: 0.01[-0.28,0.30]; CG: -0.13[-0.44,0.18] |
|  | **WMS-R Visual Memory Span Forward** | AT: -0.06[-0.37,0.25] | AT: -0.06[-0.37,0.25]; RT: 0.27[-0.04,0.58]  CT: -0.02[-0.33,0.29]; CG: -0.11[0.42,0.20] |
|  | **WMS-R Digit Span Backward** | AT: 0.25[-0.02,0.52] | AT: ;0.25[-0.02,0.52]; RT: 0.22[-0.05,0.49]  CT: 0.04[-0.23,0.31]; CG: 0.17[-0.10,0.44] |
|  | **WMS-R Visual Memory Span Backward** | AT: -0.02[-0.31,0.27] | AT: -0.02[-0.31,0.27]; RT: 0.30[0.01,0.59]  CT: 0.39[0.10,0.68]; CG: 0.20[-0.09,0.49] |
|  | **Verbal Fluency**  **Category Fluency Test** | AT: 1.28[0.36,2.20] | AT: 1.28[0.36,2.20]; RT: 0.32[-0.60,1.24]  CT: 0.97[0.09,1.85]; CG: 1.22[0.30,2.14] |
|  | **Verbal Fluency**  **Letter Fluency Test** | AT: 0.04[-0.65,0.73] | AT: 0.04[-0.65,0.73]; RT: 0.13[-0.56,0.82]  CT: -0.18[-0.85,0.49];CG: -0.003[-0.69,0.69] |
|  | **Processing Speed**  **WAIS-III Digit Symbo** | AT: 3.02[1.55,4.99] | AT: 3.02[1.55,4.99]; RT: 4.15[2.66,5.64]  CT: 1.21[-0.20,2.62]; CG: 3.94[2.45,5.43] |
|  | **Processing Speed**  **Stroop Test Color** | AT: 0.03[-0.70,0.76] | AT: 0.03[-0.70,0.76]; RT: -0.30[-1.03,0.43]  CT: -0.19[-0.9,0.52]; CG: 0.52[-0.21,1.25] |
|  | **Processing Speed**  **Trail Making Test part A** | AT: -2.18[-4.87,0.51] | AT: -2.18[-4.87,0.51]; RT: -4.05[-6.74,-1.36]  CT: -2.24[-4.81,0.33]; CG: -2.52[-5.22,0.18] |
|  | **Executive Function**  **Stroop Test Colored Word** | AT: 0.51[-2.43,3.45] | AT: 0.51[-2.43,3.45]; RT: -1.02[-3.96,1.92]  CT: 0.01[-2.79,2.81]; CG: -1.66[-4.60,1.28] |
|  | **Executive Function**  **Trail Making Test part B** | AT: -8.38[-16.73,-0.03] | AT: -8.38[-16.73,-0.03]; RT: -4.76[-13.14,3.56]  CT: 1.03[-6.99,9.05]; CG: 1.69[-10.06,6.68] |
| Silva et al.,2025, Portugal [50] | **MOCA** | ATCT: Baseline 24.1 ± 2.6; 16 weeks: 26.1 ± 2.7^d^ | ATCT: 26.1 ± 2.7; STCT: 24.1 ± 4.5^d^  ST: 23.9 ± 3.9^d^; AT: 25.7 ± 2.1^d^ |
| Song et al.,2019,China [51] | **MOCA** | IG: Baseline 22.03±1.81; 16 weeks 23.66±1.92^d^ | IG: 23.66±1.92; CG: 21.40±2.27^d^ |
| Chen et al.,2023, China [52] | **MoCA Score** | Tai chi chuan group:  Baseline 21.38±2.77; 24weeks 23.99±3.10;  36 weeks 24.67±2.72^b^ | Tai chi chuan group:  24 weeks: 23.99±3.10; 36 weeks: 24.67±2.72  Fitness walking group:  24 weeks: 23.55±3.34; 36 weeks: 23.84±3.17^b^  CG:  24 weeks: 22.54±2.29^b^; 36 weeks: 22.77±3.29^b^ |
|  | **MQ** | Tai chi chuan group:  baseline 90.04±13.69; 24weeks 98.85±10.41  36 weeks 99.39±12.70^b^ | Tai chi chuan group:  24 weeks: 98.85±10.41; 36 weeks: 99.39±12.70  Fitness walking group:  24 weeks: 95.89±13.28; 36 weeks: 95.14±14.37^b^  CG:  24 weeks: 92.79±14.13b; 36 weeks:92.98±14.43^b^ |
|  | **DSST(digit Symbol Substitution test score)** | Tai chi chuan group:  Baseline 29.53±9.91; 24weeks 34.14±10.79  36 weeks 33.82±10.48^b^ | Tai chi chuan group:  24 weeks: 34.14±10.79; 36 weeks: 33.82±10.48  Fitness walking group:  24 weeks: 30.82±9.67^b^; 36 weeks: 32.05±9.89  CG:  24 weeks: 29.81±9.85^b^; 36 weeks: 30.70±9.60 |
|  | **TMT-Bfinding** | Tai chi chuan group:  Baseline 224.41±95.16; 24weeks 202.25±72.19; 36 weeks 187.76±74.46 | Tai chi chuan group:  24 weeks: 202.25±72.19; 36weeks:187.76±74.46  Fitness walking group:  24weeks: 206.78±75.11; 36weeks: 206.62±77.65  CG:  24weeks: 223.86±85.00; 36weeks:215.53±74.47^b^ |
|  | **Boston Naming Test score** | Tai chi chuan group:  Baseline 22.46±3.21; 24weeks 24.73±2.73  36 weeks 25.37±2.42^b^ | Tai chi chuan group:  24 weeks: 24.73±2.73; 36 weeks: 25.37±2.42  Fitness walking group:  24 weeks: 24.86±2.97; 36 weeks: 25.30±3.34  CG:  24 weeks: 24.04±3.54; 36 weeks: 24.73±3.63 |
|  | **Rey-Osterrieth Complex Figure test score** | Tai chi chuan group:  Baseline 32.21±5.90; 24weeks 33.01±5.53  36 weeks 32.76±6.25 | Tai chi chuan group:  24 weeks: 33.01±5.53; 36 weeks: 32.76±6.25  Fitness walking group:  24 weeks: 32.30±5.62; 36 weeks: 31.74±6.63  CG:  24 weeks: 32.68±3.89; 36 weeks: 32.36±5.00 |
|  | **Rey-Osterrieth Complex Figure test delay**  **recall score** | Tai chi chuan group:  Baseline 13.81±7.71; 24weeks 17.90±8.21  36 weeks 18.97±8.35 | Tai chi chuan group:  24 weeks: 17.90±8.21; 36 weeks: 18.97±8.35  Fitness walking group:  24 weeks: 18.26±8.01; 36 weeks: 18.91±8.15  CG:  24 weeks: 18.16±8.44; 36 weeks: 18.87±8.53 |
| ^a^: Indicates statistical significance compared with pretraining at p=0.001;  ^b^: Indicates statistical significance compared with pretraining at p<0.05  ^c^: Indicates statistical significance compared with pretraining at p=0.01; ^d^: Indicates statistical significance compared with pretraining at p<0.01 | | | |

**Table S3** Risk of bias of included studies

| **Interventional study** |  |  |  |  |  |  |  |  |  |  |  |  |
| --- | --- | --- | --- | --- | --- | --- | --- | --- | --- | --- | --- | --- |
| **Randomized controlled trial** | **C1** | **C2** | **C3** | **C4** | **C5** | **C6** | **C7** |  |  |  |  |  |
| Choi et al., 2018 [37] | + | + | - | + | + | + | + |  |  | + | ? | - |
| Krootnark et al., 2024 [38] | + | + | - | + | + | + | + |  |  |  |  |  |
| Suzuki et al., 2013 [39] | + | + | - | + | + | + | + |  |  |  |  |  |
| ten Brinke et al., 2015 [40] | + | + | - | + | - | + | + |  |  |  |  |  |
| Zotcheva et al., 2022 [41] | + | + | - | + | + | + | + |  |  |  |  |  |
| Wang et al., 2019[42] | + | ? | - | - | + | + | ? |  |  |  |  |  |
| Xu et al., 2023 [43] | + | - | - | - | + | + | + |  |  |  |  |  |
| Yao et al., 2024 [44] | ? | - | - | - | + | + | + |  |  |  |  |  |
| Choi et al., 2019 [45] | + | + | - | + | + | + | + |  |  |  |  |  |
| De Sá et al., 2024 [46] | + | + | - | + | - | - | ? |  |  |  |  |  |
| Hsu et al., 2018 [47] | + | + | - | + | ? | + | ? |  |  |  |  |  |
| Li et al., 2023 [48] | + | + | - | + | + | + | + |  |  |  |  |  |
| Makino et al., 2021 [49] | + | + | - | + | + | + | + |  |  |  |  |  |
| Silva et al., 2025 [50] | + | ? | - | ? | + | + | + |  |  |  |  |  |
| Song et al., 2019 [51] | + | + | - | + | + | + | + |  |  |  |  |  |
| Chenet al., 2023 [52] | + | + | - | + | + | + | + |  |  |  |  |  |

C1: Random sequence generation; C2: Allocation concealment; C3: Blinding of participants and personnel; C4: Blinding of outcome assessment; C5: Incomplete outcome data; C6: Selective reporting; C7: Other bias.

Table S4 Description of exercise intensity

| **Author, year, country** | **Exercise Intervention(s)** | **Intensity as described in study** | **Intensity definition/ moitoring method** | **Was intensity compared ?** |
| --- | --- | --- | --- | --- |
| Choi et al., 2018, South Korea [37] | IG: Ground Kayak Paddling Exercise  twice a week for 6 weeks on  the ground, and each session consisted of 10 min of warm-up  activities, 40 min of GKP exercise, and 10 min of cool-down  CG: home exercise program  twice a week for 6 weeks and received a weekly confirmation  call from the instructor | Not specified | Not reported | NO |
| Krootnark et al., 2024, Thailand [38] | **The aerobic exercise group:**  Low impact exercise:  indoor walk, march in place, step in difference directions  **The resistance exercise**  **group:**  Shoulder flexion, abduction, elbow flexion, extension, hip extension,  abduction, Knee extension, plantar flexion, wall push up, step ups  **CG:**  continue their usual daily life activities with the exception of engaging in any type of exercise or cognitive training until the end of the study | low-intensity | Yes. Intensity defined and monitored using the Borg Rating of Perceived Exertion (RPE) Scale (6-20). Participants were instructed to maintain an exhaustion level of ≤13 points ("somewhat hard") and between 9–13 to ensure low intensity. | Yes (Aerobic vs. Resistance vs. Control) |
| Suzuki et al., 2013, Japan [39] | IG: biweekly 90-minute sessions involving aerobic exercise, muscle strength training, postural balance retraining, and dual-task training, a focus on promoting exercise and behavior change  CG: two education classes about health promotion involving healthy diet, oral care, prevention of urinary incontinence, and health checks | Approximately 60% of maximum heart rate | Partially. Intensity for aerobic exercise was defined as 60% HRmax, similar to previous studies. No specific monitoring method mentioned for each session. Other components (strength, balance) did not have intensity defined. | No (Exercise vs. Education Control) |
| Ten Brinke et al., 2015, Netherlands [40] | RT：Keiser Pressurised Air system(biceps curls, triceps extension, seated row, latissimus dorsi pull downs, leg press, hamstring curls and calf raises) + other key strength exercises included mini-squats, mini-lunges and lunge walks.  AT: outdoor walking programme  BAT：stretching exercises, range of motion exercises, balance exercises, functional and relaxation techniques.  60 min in duration daily | AT: 40% HRR (heart rate reserve), progressed to 70–80% HRR over 12 weeks.  RT: 6-8 reps (two sets), RPE 13-15.  BAT: Not specified. | Yes. AT: Heart rate monitors, Borg Rating of Perceived Exertion (Borg’s RPE )  (target 13-15), and ‘talk ’test.  RT: Borg RPE (target 13-15).  BAT: No intensity monitoring. | Yes (AT vs. RT vs. BAT) |
| Zotcheva et al.,2022, Norway [41] | MICT: continuous training consisted of 50 min of continuous aerobic exercise at moderate  intensity (70% of peak heart rate), corresponding to ~ 13 on  the Borg scale for ratings of perceived exertion almost daily , or to two weekly sessions of aerobic  exercise over 5 years.  HIIT: comprised ~ 40 min of interval training, consisting of  4-min working periods at 85–95% of peak heart rate (~ 16  on the Borg scale) with 3-min active breaks (60-70%  of peak heart rate) in between twice weekly  CG: Recommended  to follow national recommendations for physical activity | MICT: "Moderate intensity" (70% of peak heart rate, ~13 on Borg scale). HIIT: "High intensity" (85–95% of peak heart rate for 4-min intervals, ~16 on Borg scale; active breaks at 60–70% of peak heart rate). | Yes. MICT & HIIT: Intensity prescribed based on percentage of peak heart rate and Borg RPE scale. Monitored via heart rate monitors during supervised spinning sessions (once every 6 weeks). Outdoor training sessions were organized but unsupervised. | Yes (MICT vs. HIIT vs. Control) |
| Wang et al., 2019,China [42] | IG: gymnastic exercises + community usual health education  CG: community usual health education | Small to moderate intensity aerobic exercise | No | No |
| Xu et al.,2023, China [43] | IG:  Routine care and health education +12-week practice of Baduanjin  CG: Routine care and health education | Target heart rate controlled at (170 – age), approximately 90–110 bpm; achieving slight sweating and a feeling of “Qi fullness” | Partially. Intensity was defined using a target heart rate formula (170–age), but no objective monitoring tools were mentioned. Participants were instructed to self-monitor based on heart rate range and subjective sensations. Adherence was tracked via exercise diaries, family supervision, and bi-weekly telephone follow-ups. | No |
| Yao et al., 2024, China [44] | IG:  Routine care and health education +Six-month Baduanjin  Combination of exercise and cup-stacking training  CG: Routine care and health education | Not specified | Not reported | NO |
| Choi et al., 2019, Republic of Korea [45] | IG:VKP Exercise  each session consisted of a 10-min warm-up,  40-min VKP exercise, and 10-min cooldown. the paddling exercise in a virtual environment for 60 min twice a week for 6 weeks,  CG: home exercise | Not specified. The exercise was described as "low resistance" (1 kg paddle) with repetitive motions. Participants were asked to "row with increased effort" to maximize effect. | Not reported | NO |
| De Sá et al.,2024, Brazil [46] | PE: a multimodal physical exercise protocol, predominantly aerobic and comprising the  work of the components of functional capacity  MT: the motor task complexity protocol  was based on the two-dimensional model of Gentile's taxonomy  PE+MT: formed with the intention of proposing  activities that addressed both interventions  Six months of intervention twice a week resulted in improvements in cognitive function, total cholesterol | Strength exercise: 40–70% of 1RM.  Aerobic exercise: 60–70% of heart rate reserve  MT: Not specified; focused on task complexity, not metabolic load. | Strength intensity defined by %1RM; aerobic intensity defined by %HRR. Monitoring methods not explicitly detailed. MT intensity not defined or monitored. | Yes (PE vs. MT vs. PE+MT) |
| Hsu et al.,2018, Canada [47] | IG: Aerobic training and compliance  thrice-weekly 60min classes of  walking for the 6-month intervention period.  CG: usual care | “Moderate-intensity” aerobic training; progressed to the range of 60–70% of heart rate reserve (HRR) | Intensity was defined using %HRR. Monitored via heart rate monitors, Borg Rating of Perceived Exertion (RPE), the "talk test," and pedometers. | No (Aerobic training vs. Usual care control) |
| Li et al., 2023, USA [48] | enhanced tai ji quan:  the standard tai ji quan +  practice in dynamic tai ji quan forms interwoven concomitantly with a set of cognitively demanding activities  standard tai ji quan:  Participants received verbal and visual instruction  cues for sequential practice of 8 tai ji quan forms  stretching :  participants received stretching exercises | Tai Ji Quan: Described as “moderate-intensity mind-body exercise” Stretching: Described as “light static stretches” with “intermittent light walking” | Partially. Intensity was qualitatively described but not defined using objective metrics. Monitoring methods not specified. | Yes (Cognitively Enhanced Tai Ji Quan vs. Standard Tai Ji Quan vs. Stretching) |
| Makino et al.,2021,Japan [49] | AT:  10–15 min of step-in-place exercises+10-15min of a walking program +intervals for rest and monitoring rate.  RT:  elastic resistance training  + bodyweight exercises  CT: combined the AT and RT programs  CG: attend educational classes 2 times during the 26-week | Target RPE: “Easy” for weeks 1–2, “Somewhat hard” for weeks 3–12, “Hard” (15) for remainder. AT: Target HRR progressed from 40% (weeks 1–2) to 70% (weeks 9–26). RT: Elastic resistance and bodyweight exercises; intensity progressed by changing grip width or rubber stiffness. | Yes. Intensity defined using RPE (Borg scale) and %HRR (for AT). Monitored via: AT: wearable heart rate devices, pedometers, exercise notebooks. RT: exercise notebooks, RPE recording. | Yes (AT vs. RT vs. CT vs. Control) |
| Silva et al.,2025, Portugal [50] | STCT，strength plus cognitive training  ST，strength training  AT，aerobic training  ATCT，aerobic plus cognitive training  The interventions were implemented over 12 consecutive  weeks, comprising 60-minute sessions conducted three  times per week, with a 48-hour interval between each session. | Aerobic: the rate of perceived exertion (RPE) 5–6/10 (warm-up), 7–8/10 (main), 3–4/10 (cool-down); heart rate (HR) 60% (warm-up), 75–85% (main), 40% (cool-down).  Strength: repetitions in reserve (RIR) 4–5; HR 75–85%. | Yes. Intensity defined using Borg RPE (0–10 scale), %HR, and Repetitions in Reserve (RIR). Load adjusted based on participants' RPE responses. | Yes (AT vs. ST vs. ATCT vs. STCT) |
| Song et al.,2019,China [51] | IG: aerobic stepping exercise programme with three 60-minute group training sessions (20 participants per group) per week  CG: 16-week health education programme | “Moderate-intensity” aerobic exercise; target Borg Rating of Perceived Exertion (RPE) 12–14 | Yes. Intensity defined using Borg RPE scale (6–20). Participants were instructed to pace their movements to achieve a feeling of “somewhat hard” (Borg 12–14). Exercise was introduced progressively, with the aerobic duration increasing from 20 to 40 minutes over 4 weeks. | No (Exercise vs. attention-placebo control) |
| Chen et al.,2023, China [52] | Tai chi chuan group: T2D management+ 24-form tai chi chuan training.  Fitness walking group: T2D management + 24-week fitness walking program.  CG: T2D management  Both exercise groups took the training for  60 min/session, 3 times/wk, for 24 weeks in a supervised setting | Tai Chi Chuan: Described as “moderate-intensity aerobic exercise”, overlapping with brisk walking.  Fitness Walking: Moderate-intensity aerobic exercise. | Partially. Intensity was qualitatively described as moderate-intensity based on MET equivalents, but no objective monitoring methods were specified. | Yes (Tai Chi vs. Fitness Walking vs. Control) |

Table S5 Cognitive task integration

| Author, year, country | Intervention type | Cognitive task integration | Description of cognitive component |
| --- | --- | --- | --- |
| **Pure aerobic interventions** | | | |
| Wang et al., 2019,China [42] | Aerobic gymnastics | / | / |
| Ten Brinke et al., 2015, Netherlands [40] | Aerobic training | / | / |
| Hsu et al.,2018, Canada [47] | Aerobic training | / | / |
| Zotcheva et al.,2022, Norway [41] | Aerobic (MICT/HIIT) | / | / |
| Song et al.,2019,China [51] | Aerobic stepping | / | / |
| Makino et al.,2021,Japan [49] | Aerobic training | / | / |
| Krootnark et al., 2024, Thailand [38] | Low-intensity aerobic | / | / |
| Sequential interventions | | | |
| Silva et al.,2025, Portugal [50] | Aerobic + cognitive training | Sequential | 20-min cognitive training (Fit4Alz software) performed after aerobic exercise. |
| Intrinsic/Embedded intervention | | | |
| Xu et al.,2023, China [43] | Baduanjin | Intrinsic | Complex, coordinated movements requiring attention and memory; no explicit additional cognitive task. |
| Yao et al., 2024, China [44] | Baduanjin + cup-stacking | Intrinsic | Combination of two complex motor tasks (Baduanjin and cup-stacking) that inherently engage cognitive processes. |
| De Sá et al.,2024, Brazil [46] | PE+MT | Intrinsic | Progressive motor skill complexity based on Gentile's taxonomy; tasks require attention and coordination but no separate cognitive training. |
| Chen et al.,2023, China [52] | Tai Chi Chuan | Intrinsic | Mind-body exercise requiring memorization, concentration, and coordination; no explicit cognitive task. |
| Choi et al., 2018, South Korea [37] | Ground kayak paddling | Intrinsic | Complex paddling movements on unstable surface; requires coordination and attention; no separate cognitive task. |
| Choi et al., 2019, Republic of Korea [45] | Virtual kayak paddling | Intrinsic | Virtual reality kayaking with directional decision-making; requires cognitive processing but no explicit secondary task. |
| **Simultaneous/Dual-task exercise** | | | |
| Suzuki et al., 2013, Japan [39] | Multicomponent exercise | Simultaneous | Dual-task training: walking while performing cognitive tasks. |
| Li et al., 2023, USA [48] | Cognitively enhanced Tai Ji Quan | Simultaneous | Tai Ji Quan integrated with concurrent cognitive tasks. |
| MICT: Moderate-intensity continuous training  HIIT: High-intensity continuous training  PE: Physical exercise; MT: The motor task complexity protocol | | | |
